# Nutraceutical Periodization for eXercise (NPX): Matching Bioactives to Training Load, Recovery Demand, and Adaptation Windows

**DOI:** 10.3390/nu18203337

**Published:** 2026-10-12

**Authors:** Dan Cristian Mănescu, Cristina Casals, Anna Kurkiewicz-Piotrowska, Tayana Silva de Carvalho, Costin Petcu, Cristian Băltărețu, Radu Bidiugan, Andrei Dragomir, Octavian Amzulescu, Simona Bidiugan, Osama Salloum, Răzvan Alexandrescu

**Affiliations:** 1 Department of Physical Education and Sport, Bucharest University of Economic Studies, 010374 Bucharest, Romania; dan.manescu@defs.ase.ro; 2 ExPhy Research Group, Department of Physical Education, Instituto de Investigación e Innovación Biomédica de Cádiz (INiBICA), 11009 Cádiz, Spain; cristina.casals@uca.es; 3 Faculty of Physiotherapy, University of Physical Culture, 31-571 Kraków, Poland; anna.piotrowska@awf.krakow.pl; 4 Herz-und Diabeteszentrum NRW, Diabeteszentrum, Ruhr-Universität Bochum, 32545 Bad Oeynhausen, Germany; carvalhotay89@gmail.com; 5 Faculty of Medicine, Carol Davila University of Medicine and Pharmacy, 050474 Bucharest, Romania; 6 Romanian Bodybuilding and Fitness Federation, 010374 Bucharest, Romania; 7 Doctoral School, National Carol Davila University of Physical Education and Sport, 060057 Bucharest, Romania; razvan.alexandrescu@unefs.ro; 8 Carol Davila National Institute for Sport Research, 060057 Bucharest, Romania; bidiugan@sportscience.ro (R.B.); octavian.amzulescu@sport-science.ro (O.A.); catanoiu@sportscience.ro (S.B.); 9 Witting CF1 Clinical Hospital, 011791 Bucharest, Romania; osama.salloum@drd.umfcd.ro; 10 Pharmacy, Science and Technology of Târgu Mureș, George Emil Palade University of Medicine, 540139 Târgu Mureș, Romania

**Keywords:** nutraceuticals, sports nutrition, dietary supplements, exercise adaptation, recovery, periodization, precision nutrition, athlete health

## Abstract

Nutraceuticals are evaluated as though efficacy were a stable ingredient property, although net value may vary with exercise stimulus, exposure schedule, recovery pressure, athlete state, and prioritized outcome. This structured narrative review integrates exercise-adaptation biology, periodized sports nutrition, training-load and recovery models, precision nutrition, and human evidence for sport-relevant interventions. Searches of PubMed/MEDLINE, Web of Science, and Scopus were updated through August 2026 and supplemented by citation tracking. General efficacy was separated from direct evidence of timing, context, or state modification. Nutraceutical Periodization for eXercise (NPX) is an allocation layer applied only after safety, legality, product quality, and plausible efficacy are established. It defines four non-sequential functional windows—performance exploitation, adaptation-permissive signaling, restoration, and resilience—and separates intervention strategy, training context, athlete state, and outcome hierarchy. Four falsifiable propositions test context modification, temporal allocation, bottleneck/state dependence, and whole-policy utility. None of the allocation claims in the present evidence map met the criterion for direct NPX allocation evidence. NPX therefore formalizes hypothetical allocation rules rather than an established supplementation policy; proposed improvements in athlete-centered outcomes or equivalent outcomes with less exposure, burden, or harm remain to be prospectively tested against an explicitly defined active evidence-based comparator. Interaction and policy trials are needed to establish the value and boundaries of these rules.

## 1. Introduction

Most supplement trials ask a necessary question: does the intervention work? Sport practice requires a second question: under what conditions is the effect worth pursuing? Athletes encounter nutrients and bioactive compounds before competition, during high-quality training, across loading blocks, after tissue-damaging work, during travel, and under changing energy availability, sleep, illness risk, and injury status. A mean effect can establish biological or practical efficacy, yet still obscure whether the intervention adds value in a particular session, block, or athlete state.

In this review, nutraceutical is used operationally as an umbrella term for food-derived bioactives, isolated food components, and dietary-supplement ingredients deployed to modify exercise performance, recovery, adaptation, or athlete health. It is not used as a regulatory category and does not imply efficacy. Any application remains subordinate to adequate energy and nutrient intake, product identity and quality, legality, contamination control, medical screening, and an evidence-based risk–benefit assessment [1,2,3,4,5].

The evidence is most persuasive when an intervention has a clear mechanism, an exposure profile that matches that mechanism, and an endpoint that expresses it. Caffeine can improve selected cognitive and exercise outcomes; dietary nitrate can support nitric-oxide availability and exercise economy; creatine can increase phosphocreatine availability and repeated high-intensity capacity; and beta-alanine can increase muscle carnosine and buffering capacity [6,7,8,9]. Their temporal profiles differ. Caffeine and nitrate can be deployed at the session scale, whereas creatine and beta-alanine generally require loading and maintenance across days or weeks. Timing therefore encompasses not only minutes before exercise, but also when exposure is initiated, maintained, restricted, or withdrawn.

Interpretation becomes more difficult when the intended outcome is adaptation, tissue remodeling, recovery, or resilience. Recent human exercise studies illustrate why a binary positive/negative label is inadequate. New Zealand blackcurrant extract altered substrate storage and intramuscular triglyceride use without improving exercise capacity; acute nutritional ketosis did not alter the early skeletal-muscle transcriptomic response to aerobic exercise; and a betalain-rich concentrate affected heart rate, perceived exertion, and recovery muscle oxygenation without improving running economy or maximal oxygen uptake [10,11,12]. Curcumin did not accelerate recovery after a simulated soccer match, green tea did not augment the expected Nrf2 response after eccentric exercise, and bovine colostrum improved lower-body power in rugby players without clearly improving the selected immune, inflammatory, or illness outcomes [13,14,15]. Such findings can indicate limited efficacy, outcome mismatch, or genuine context dependence. Only prospectively designed comparisons can distinguish these explanations.

This review introduces Nutraceutical Periodization for eXercise (NPX) as a framework for deciding whether an already evidence-eligible strategy should be initiated, maintained, shifted, or withdrawn as the dominant training task and athlete state change. *Intervention strategy* denotes the specified product, formulation, dose, and exposure schedule. *Functional allocation window* denotes a prospectively assigned decision state in which one physiological task is prioritized; it is not a fixed clock interval or a claim of a discrete biological compartment. *Athlete state* denotes a pre-exposure characteristic that may modify response. The multiplication sign is reserved in this article for an estimable statistical interaction, not used as shorthand for conceptual association.

Periodized nutrition aligns nutrient and fuel availability with training demands; precision nutrition characterizes individual needs and responses; recovery frameworks organize restoration priorities; and supplement classifications establish product eligibility [1,16,17,18,19,20,21,22,23,24,25,26,27,28]. Training–Fuel Coupling [29] and Recovery–Adaptation Coupling [30] are related conceptual antecedents, including goal-dependent timing and bottleneck-based decisions. NPX extends this territory by specifying strategy-level initiation, maintenance, shifting, withholding, and exit decisions across four functional windows, separating efficacy from allocation evidence, and defining matched component contrasts and whole-policy tests. Its proposed novelty lies in this integrated evaluation architecture; its practical incremental value remains untested.

The synthesis was organized around four research questions:

**RQ1.** What decision construct does NPX formalize beyond existing evidence classifications, periodized-nutrition models, recovery frameworks, and precision-nutrition approaches?

**RQ2.** For an evidence-eligible strategy, does the magnitude or direction of its effect differ across prospectively defined training contexts or exposure schedules when product, dose, cumulative exposure, adherence, and carryover are adequately controlled?

**RQ3.** Does an independently defined pre-exposure bottleneck or athlete-state characteristic modify the effect on a prioritized athlete-centered outcome?

**RQ4.** Does a locked NPX-guided allocation policy improve the prioritized athlete-centered outcome—or preserve it with fewer exposures, lower burden, or less harm—relative to standardized evidence-based use?

Accordingly, the objectives were to define the functional allocation window as a decision unit; position NPX relative to adjacent frameworks; organize representative interventions across performance-exploitation, adaptation-permissive, restoration, and resilience priorities; and derive propositions, estimands, and study designs capable of supporting, delimiting, or rejecting the framework.

## 2. Narrative Review Methodology

### 2.1. Design, Scope, and Analytical Framework

This article was designed as a structured narrative review and theory-building synthesis rather than a systematic review or meta-analysis. This design was selected because the question spans heterogeneous evidence streams—exercise-adaptation biology, supplement efficacy and kinetics, training-load and recovery models, athlete-state modifiers, and periodized nutrition—and because the principal aim was to derive prospectively testable relationships rather than a pooled treatment effect [31,32,33,34]. Explicit information sources, eligibility criteria, appraisal domains, and synthesis rules were nevertheless used to reduce selective interpretation. The review does not claim exhaustive retrieval, formal certainty grading, or quantitative estimates of efficacy.

The population of primary interest comprised competitive athletes and exercise-trained adults. Evidence from healthy non-athlete adults was retained when needed to characterize pharmacokinetics, tissue exposure, mechanism, or a controlled exercise response that could not be isolated adequately in athletes. Evidence from clinical, injured, immobilized, or deficient populations was used only to inform the corresponding state-dependent mechanism or boundary condition and was not treated as direct evidence of efficacy in healthy athletes.

The intervention scope comprised protein and amino acids, creatine, caffeine, dietary nitrate, sodium bicarbonate, beta-alanine, polyphenols and isolated antioxidants, *n*-3 polyunsaturated fatty acids (*n*-3 PUFA), probiotics, bovine colostrum, medically indicated micronutrient correction, and collagen-derived peptides. Energy and carbohydrate availability were treated as foundational conditions rather than as window-allocated nutraceutical interventions. One human exercise study of acute nutritional ketosis [11] was retained only to illustrate discordance between a mechanistic endpoint and a practical allocation claim; ketone interventions were not mapped as NPX candidates. Eligible comparators included placebo or no intervention, an active comparator, the same cumulative exposure delivered at a different time or in a different training context, and prospectively defined athlete states with and without the proposed bottleneck.

Outcomes were classified into three roles before interpretation: (i) one prioritized athlete-centered outcome, such as performance, session completion, recovery of function, illness days, gastrointestinal symptoms, or missed training; (ii) constraint outcomes representing potential costs, such as sleep disruption, gastrointestinal intolerance, symptom burden, body-mass change, impaired adaptation, or adverse events; and (iii) mechanistic outcomes used to examine biological coherence. A mechanistic change alone was not considered evidence of practical benefit.

### 2.2. Information Sources and Search Strategy

PubMed/MEDLINE, Web of Science Core Collection, and Scopus were searched iteratively during manuscript development, with the final update undertaken in August 2026. Searches were supplemented by backward reference checking and forward citation tracking from consensus statements, position stands, systematic reviews, mechanistic reviews, and influential athlete studies. Transparent final search templates were retained, while the iterative process did not generate a PRISMA-style audit trail.

Three concept blocks were used: (A) exercise or athlete context; (B) nutraceutical, supplement, bioactive, or named intervention; and (C) allocation, timing, training context, athlete state, or outcome. Framework-level searches combined A AND B AND C. Because requiring timing- or context-related language could preferentially retrieve studies already framed around periodization, a second efficacy-and-safety route combined block A with block B—or its named-intervention substitute—and an evidence-type anchor comprising systematic review, meta-analysis, consensus, position statement, or position stand, without requiring block C. Compound-specific searches replaced the broad intervention block with the relevant named-intervention expression and, where applicable, retained the subset of block C appropriate to the intervention’s kinetics, proposed moderator, or endpoint. The exact executable strings, concept definitions, search-field implementation, efficacy-and-safety route, and compound-specific substitution rules are reported in Supplementary Method S1 and Table S1.

The final search architecture contained no lower publication-date bound and no common language or document-type restriction. This does not establish database-inception coverage or uniform filters across historical exploratory queries: interface-specific filter logs and day-level timestamps were not retained. Comprehensive non-English retrieval or translation is not documented and is therefore not claimed. The final cited corpus uses peer-reviewed sources for efficacy claims and excludes commercial, promotional, and unreviewed claims as specified in Section 2.3; no uniform historical publication-status filter can be reconstructed. Supplementary Method S1 and Table S1 distinguish these reporting limits from the final executable search templates.

### 2.3. Eligibility and Evidence Selection

Evidence was retained when it met all of the following criteria: (i) it addressed an intervention within scope, apart from the single endpoint-discordance exemplar identified in Section 2.1 [11]; (ii) it involved an exercise, training, competition, recovery, injury/disuse, illness-risk, or athlete-relevant physiological context; (iii) it reported an athlete-centered, constraint, kinetic, exposure, or mechanism-linked outcome relevant to at least one research question; and (iv) it contributed to general efficacy and safety, exposure time course, context or state dependence, responder heterogeneity, or a plausible trade-off among performance, recovery, adaptation, and health.

Peer-reviewed consensus or position statements, systematic reviews and meta-analyses, randomized human trials, longitudinal training studies, and controlled mechanistic human experiments received priority. Seminal animal, tissue, or cell evidence was retained only when required to establish biological plausibility not available from human research and was not used to override neutral or adverse athlete-centered findings. Commercial or promotional material, unreviewed claims, duplicate reports, inadequately characterized multi-ingredient products, studies without a meaningful exercise- or athlete-state link, and reports that did not permit the intervention, exposure, comparator, or relevant outcome to be identified were excluded from efficacy claims.

Search outputs and citation-tracking records were examined against these criteria during iterative synthesis, and full texts were consulted for retained claims. Because records were not managed under a prospectively specified systematic-review protocol, numerical screening counts and article-level exclusion logs were not retained, independent duplicate screening is not claimed, and a PRISMA flow diagram was not constructed. The resulting corpus was evaluated across the stated appraisal domains rather than treated as an exhaustive census of the literature.

Methodology and literature evaluation were collective author contributions, as recorded in the Author Contributions statement. The narrative-review documentation does not identify task-specific personnel for each database query, eligibility decision, extraction step, or qualitative appraisal, and no adjudication log was retained. A formal disagreement-resolution procedure cannot therefore be reconstructed. Collective author responsibility should not be interpreted as independent duplicate screening, extraction, or coding; none is claimed. These reporting limitations constrain the reproducibility of the historical review process.

### 2.4. Evidence Appraisal and Data Organization

Formal risk-of-bias scoring and certainty grading were not undertaken. Each source was instead appraised qualitatively across six domains: study design; population relevance; intervention and product characterization; exposure and temporal fit; validity and proximity of the outcome to the claim; and consistency with higher-level human evidence. Interpretation also considered sample size and precision when reported, adherence, blinding credibility, control of diet and training load, carryover or washout, and ecological validity. Greater weight was assigned to convergent systematic reviews and well-characterized randomized human studies with outcomes aligned to the claim. Mechanistic or preclinical evidence was used to explain plausibility, not to rescue an efficacy claim contradicted by human athlete-centered outcomes.

The evidence map organizes the stated intervention, candidate decision role, efficacy and allocation-directness code, source citations, temporal or state features, and permissible inference. It is a qualitative claim-level organization of the retained literature rather than an independently duplicated extraction dataset.

To prevent distinct evidentiary questions from being collapsed, each candidate claim is interpreted on two orthogonal axes. Efficacy status applies to an explicitly bounded product or intervention, population, comparator, and athlete-relevant outcome, rather than to an ingredient universally. E denotes convergent human comparative evidence of practical efficacy: a consistent evidence synthesis and/or independently replicated, adequately characterized comparative studies with athlete-centered outcomes support the defined claim without unresolved major contradictory findings. C denotes human comparative evidence relevant to the practical claim, but with limited replication, inconsistent findings, imprecision, product or protocol heterogeneity, or extrapolation beyond the tested population or outcome. M denotes a proposed practical use supported chiefly by mechanism, kinetics, preclinical findings, or human surrogate outcomes, without convincing comparative evidence for the corresponding athlete-centered benefit. A narrow task or product scope does not itself lower a code; the code concerns support for that exact scoped claim. Mechanistic changes cannot elevate a claim to E, and neutral or adverse athlete-centered results remain explicitly visible rather than being treated as evidence of efficacy.

Allocation directness is classified as D only when a prospective human comparison estimates the named timing-, context-, or state-dependent allocation contrast. For a context or state claim, this requires the relevant intervention-by-context/state contrast rather than benefit within a single context or state; for a timing claim, it requires a prespecified between-schedule comparison with adequately comparable exposure. I denotes an allocation inferred from general efficacy, kinetics, mechanism, a study conducted in only one candidate context or state, or comparisons across different studies. D indicates that the allocation contrast is estimable, not that it is meaningfully non-zero or supports NPX; E indicates efficacy within a defined scope, not validation of an allocation rule. Borderline or conflicting efficacy findings warrant a conditional or hypothesis-generating interpretation and an explicit explanation of the uncertainty. These qualitative labels are not formal risk-of-bias or certainty grades, and independently replicated coding or inter-rater reliability is not claimed. The intervention-level evidence map is provided in Supplementary Table S2.

### 2.5. Narrative Synthesis and Framework Development

Synthesis proceeded in five stages. First, recurring physiological tasks within periodized training were grouped into performance exploitation, adaptation-permissive signaling, restoration, and resilience. Second, each intervention class was mapped by general efficacy, target mechanism, onset and persistence, loading or washout requirements, candidate state modifiers, athlete-centered outcomes, and plausible constraints. Third, general efficacy was separated from direct evidence that timing, context, or state modifies efficacy. Fourth, null, mixed, and discordant findings were examined without assuming that a timing mismatch explained them. Fifth, the incremental NPX layer was stress-tested by specifying observations that would make it unnecessary or incorrect.

This process generated four research questions and four falsifiable propositions. Component propositions concern a prespecified intervention-by-context, intervention-by-timing/schedule, or intervention-by-state contrast; the framework-level proposition concerns a between-policy contrast. A full intervention × functional-window × athlete-state interaction is appropriate only when all factors are crossed, and the study is powered for that estimand. Otherwise, two-way component tests should not be described as evidence for a three-way interaction.

### 2.6. Standards for Interpretation and Prospective Testing

Each proposed NPX test requires prospective definition of the intervention, target window or context, athlete-state variable, prioritized outcome, constraint outcomes, smallest effect of interest or equivalence/non-inferiority margin, and rejection criterion. Interaction claims must be based on the interaction contrast itself rather than significance in one subgroup and non-significance in another. Continuous state variables should remain continuous where possible; a threshold used to define a bottleneck should be justified independently of the treatment response.

Repeated observations require models that account for within-athlete correlation and, where relevant, team or site clustering. Randomized comparisons should target a treatment-policy estimand analyzed according to the intention-to-treat principle, with per-protocol analyses treated as sensitivity analyses. Confidence intervals should be interpreted against a prespecified smallest effect of interest, equivalence margin, or non-inferiority margin; an underpowered nonsignificant result is inconclusive rather than evidence of invariance. Primary outcomes, directional contrasts, multiplicity procedures, missing-data handling, exposure verification, carryover assessment, and adverse-event rules should be registered before recruitment. Detailed exemplar designs and a protocol/reporting checklist are supplied in Supplementary Tables S3 and S4.

## 3. The NPX Framework: Scope, Constructs, and Operationalization

### 3.1. From Ingredient Efficacy to Allocation Value

Traditional supplement evaluation appropriately emphasizes an average intervention effect. That model becomes incomplete when the desired outcome is a training adaptation or when the cost of an intervention depends on context. Exercise is not merely a stressor to be neutralized; it is an information signal. Glycogen availability, calcium flux, mechanical tension, local hypoxia, redox perturbation, amino-acid availability, and inflammatory signaling converge on pathways that include AMP-activated protein kinase (AMPK), mechanistic target of rapamycin complex 1 (mTORC1), peroxisome proliferator-activated receptor gamma coactivator 1-alpha (PGC-1α), nuclear factor erythroid 2-related factor 2 (Nrf2), and satellite-cell-related processes [35,36,37,38]. An intervention can therefore act as a performance amplifier, signal modulator, restoration aid, or resilience buffer according to the event and state it encounters.

This reasoning is consistent with systems biology and network physiology, in which organism-level behavior emerges from interactions among components rather than isolated parts [39,40]. NPX applies this logic while preserving ingredient-specific evidence.

Periodized nutrition has operationalized related principles most clearly for substrate availability. Carbohydrate availability can support high-quality work or be strategically reduced around selected sessions to modify acute oxidative signaling, although superior longitudinal performance is not consistently established [16,17,41,42,43]. Supplement-evidence, integrated-periodization, and recovery-nutrition frameworks organize product eligibility, training demands, and recovery priorities [18,44,45,46]. Training-load and recovery models distinguish external stimulus, internal response, fatigue, and readiness [19,47,48,49]. Precision nutrition and precision exercise medicine emphasize phenotype, diet, microbiome, behavior, and response variability [20,21,22,23,24]. The conceptual comparison in Section 1 identifies how NPX proposes to extend these traditions.

### 3.2. What NPX Adds—And What It Does Not

NPX begins only after a strategy has passed the inherited gates of safety, legality, product identity and quality, and plausible compound-specific efficacy. It does not replace systematic reviews, consensus statements, position stands, clinical assessment, or dosing guidance [1,44]. A weak or contaminated product cannot be rescued by favorable timing. Nor is NPX an argument for supplement accumulation. When energy availability, carbohydrate or protein intake, sleep, training design, or medical care is the primary limitation, adding a bioactive is unlikely to solve the problem.

Table 1 operationalizes the allocation policy introduced in Section 1 through six decisions: dominant objective, pretreatment bottleneck, exposure schedule, outcome hierarchy, interpretation of discordance, and exit or reassignment. These decisions are intended to make an allocation claim testable; they do not establish that the proposed policy improves on existing practice.

Net value is therefore a qualitative decision construct: change in the prespecified priority outcome after accounting for constraints, adverse effects, interference with another objective, burden, cost, anti-doping risk, and feasibility. It is not a universal numerical score. Table 1 distinguishes the NPX increment construct by construct.

### 3.3. Four Functional Windows and Temporal Scales

A functional allocation window is a prospectively assigned decision state, not a discrete physiological compartment or a fixed number of hours. Functional windows can overlap. The dominant window identifies the outcome whose failure would most compromise the next high-priority objective; the other objectives become constraints. *Restoration* refers to recovery of function after a realized stressor, injury, or disuse, whereas *resilience* refers to prospective preservation of training availability during a defined illness, gastrointestinal, travel, or environmental risk state.

The performance-exploitation window is assigned when immediate task quality or competition performance dominates. The adaptation-permissive window is assigned when preservation of exercise-induced signaling and longitudinal remodeling dominates. The restoration window is assigned when realized loss of function threatens the next high-value exposure. The resilience window is assigned when a prospective illness, gastrointestinal, environmental, deficiency, or availability risk threatens training continuity.

The four windows share a decision structure but are not symmetric in the action they imply. Performance, restoration, and resilience often motivate administering an eligible strategy; adaptation-permissive signaling may instead motivate intentional non-initiation, restriction, rescheduling, pausing, or withdrawal when exposure could constrain the prioritized adaptation. These are formal NPX allocation actions when the eligible strategy, rationale, start/stop conditions, and outcome hierarchy are specified prospectively. Non-use is therefore an active policy choice, not evidence that a particular withholding or withdrawal schedule is effective. Clinically indicated treatment, deficiency correction, and dietary adequacy remain governed by their established indications and should not be withheld merely to preserve an adaptation window.

Window assignment concerns the intended function and timing of an effect, not necessarily clock time of ingestion [6,7,9,50]. Session-scale agents may be positioned around a bout; loading agents are periodized by initiation, maintenance, tapering, or withdrawal across a block; and state-triggered strategies are activated only when the relevant vulnerability is present. Residual exposure matters because biological activity can persist after the nominal window closes, and continuous use can turn a targeted intervention into a background condition. Exercise clock time and chronotype are additional contextual variables considered in exercise-timing frameworks [51].

**Figure 1:**
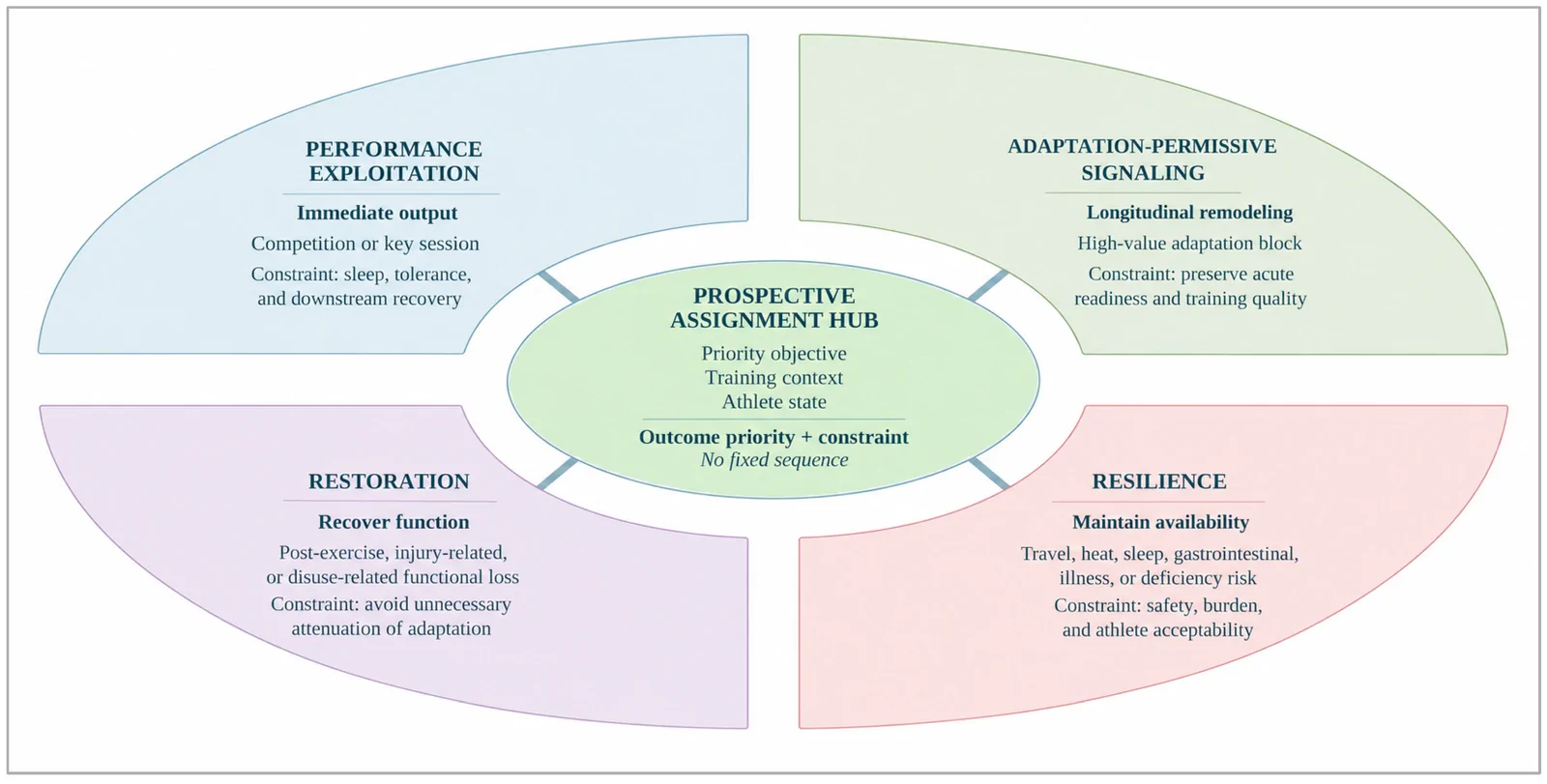
Functional allocation windows within Nutraceutical Periodization for eXercise (NPX). The four windows are non-sequential decision states defined by the dominant physiological objective rather than by fixed clock intervals. The dominant window is assigned prospectively from the priority objective, training context, and athlete state; the remaining objectives become constraint outcomes. Restoration addresses realized post-exercise, injury-related, or disuse-related functional loss, whereas resilience addresses prospective threats to training availability arising from prespecified travel, environmental, sleep, gastrointestinal, illness, or deficiency-related risk states. The contexts shown are illustrative, not prescriptive.

Figure 1 summarizes the four non-sequential functional allocation windows and the variables used to assign the dominant window prospectively.

### 3.4. Prospective Assignment and the Decision Loop

Assignment begins with a protocol-specific decision sheet completed before exposure: the next priority event or training-block endpoint and its date; the minimum acceptable performance, function, or availability needed to complete it; expected load and time to that exposure; and reliably measured pre-intervention athlete-state indicators. Clinical contraindications and safety decisions take precedence outside the allocation rule. Performance exploitation is selected when immediate task output is the locked primary objective, and the prespecified function and availability constraints are satisfied. Restoration is selected when an observed functional deficit crosses a predefined threshold and threatens completion of the next priority exposure. Resilience is selected when a predefined prospective illness, gastrointestinal, environmental, or availability-risk criterion is crossed before functional loss has occurred. Adaptation-permissive signaling is selected when a longitudinal remodeling endpoint is the primary objective and neither realized functional loss nor prospective availability risk crosses the protocol’s constraint thresholds. Cutoffs must be justified from the sport, measurement reliability, clinical criteria where appropriate, and the demands of the scheduled objective; NPX supplies no universal numerical cutoffs.

When criteria overlap, a prespecified priority order linked to the next scheduled objective determines which qualifying endpoint takes precedence; the remaining endpoints become constraints. The order and tie-break rule must be locked before treatment and cannot be derived from the response. If the inputs do not distinguish a dominant objective, the case is recorded as unresolved or overlapping rather than forced into a unique category. Reproducibility could be evaluated prospectively by having independent assessors classify identical pre-exposure case records without access to treatment outcomes, reporting category-specific and overall agreement, chance-corrected agreement with confidence intervals, and sensitivity to reasonable threshold changes. This would test classification reliability; outcome prediction and policy trials would still be required to establish decision value.

Table 2 provides a prospective rubric. It is deliberately product-agnostic: compound-specific dosing, contraindications, clinical oversight, and anti-doping responsibilities remain separate requirements.

Two examples illustrate the hierarchy. After a damaging match 36–48 h before a championship event, restoration dominates because functional loss threatens the next priority event; adaptation remains a constraint, and any short-term recovery strategy exits after the event. During a high-intensity session undertaken while traveling, performance can dominate below a prespecified gastrointestinal/illness-risk threshold; resilience becomes dominant only when that threshold indicates a credible risk of losing training availability.

## 4. Evidence Synthesis Across Four Functional Windows

Across the strategies mapped in Supplementary Table S2, none met the D criterion for a direct test of its named NPX allocation rule. All mapped placements therefore have indirect allocation status (I), even when the intervention has established efficacy (E) for a particular task or outcome. The following synthesis supports the eligibility and biological plausibility of candidate strategies; it does not demonstrate the superiority of their proposed NPX window allocation. This statement is limited to the mapped claims and does not imply that sports nutrition lacks all evidence on timing or individualization.

### 4.1. Performance Exploitation

The performance-exploitation window is dominant when the immediate objective is to increase the value of a training session or competition. A strategy is justified when it enables more work, greater velocity, improved pacing, higher neuromuscular output, or better decision-making without unacceptable costs. Caffeine, nitrate, sodium bicarbonate, creatine, and beta-alanine are representative examples, but their kinetics differ substantially. Caffeine, nitrate, and sodium bicarbonate may be used acutely or in short protocols; creatine and beta-alanine generally require sustained loading before benefits are expressed during repeated high-intensity efforts or prolonged buffering demands [6,7,8,9,52,53,54,55,56].

Sodium bicarbonate illustrates why the intervention is more than the ingredient. Benefit depends on the target task, dose, administration schedule, and individual tolerance. Gastrointestinal symptoms can themselves impair performance and vary within and between athletes. Protocols should therefore be rehearsed away from competition, with attention to the lowest evidence-supported dose, ingestion schedule, accompanying sodium load, and medical contraindications [56].

NPX links an acute ergogenic effect to the value of the session in which it occurs. Caffeine before a low-priority recovery session may add stimulant exposure without improving the training plan, whereas an acute agent before a high-priority session may improve completed work. For a block-level claim, the proposed causal chain should be explicit: exposure improves session quality; session quality alters accumulated training; and accumulated training contributes to the targeted adaptation. An acute competition benefit is a separate estimand and does not require mediation through accumulated training.

A performance window also requires a cost model. Caffeine can disturb sleep and provoke anxiety in susceptible athletes, especially when dose and time of day are poorly matched; attenuation with habitual use is inconsistent and should be measured rather than assumed [6,52]. Nitrate responses vary with oral-microbiome activity, habitual nitrate intake, exercise mode, and participant characteristics [8,54]. Creatine-related mass gain can be useful in strength or collision sports but undesirable in selected endurance settings [7,53]. Beta-alanine is unlikely to matter when the task does not challenge intracellular buffering [9,55]. Findings in resistance-trained women further caution against untested transfer from male-dominated cohorts [57]. The allocation hypothesis is that selective deployment improves net value; established acute efficacy alone does not prove that policy.

### 4.2. Adaptation-Permissive Signaling

The adaptation-permissive window asks when restraint may be the most appropriate intervention. Exercise-induced reactive oxygen species and inflammatory signals are not merely damage products; they participate in mitochondrial biogenesis, antioxidant-enzyme regulation, immune remodeling, and tissue adaptation [58,59]. Human trials of chronic high-dose isolated vitamin C and/or E have reported attenuation of selected molecular responses, and one trial reported impaired endurance-training adaptation [59,60]. By contrast, Paulsen et al. observed attenuated cellular markers without a clear reduction in maximal oxygen uptake or running-performance gains [61].

These studies tested daily exposure across training periods; they did not randomize an identical dose to proximal versus distal schedules around exercise. They justify caution with routine chronic high-dose isolated antioxidant regimens when oxidative remodeling is prioritized, but do not establish that near-session ingestion is more harmful than the same exposure taken farther from exercise. Temporal proximity remains a prospective NPX hypothesis [58,59,60,61]. These findings should not be generalized to antioxidant-rich whole foods or every polyphenol-containing product.

Related Redox-Adaptive Periodization concepts frame the distinction between signal preservation and symptom suppression [62]. This window concerns restricting or rescheduling a strategy that may be eligible for another objective; it cannot admit an unsupported antioxidant product through the evidence gate. During a block targeting mitochondrial or redox adaptation, routine high-dose isolated antioxidant supplementation is a potential constraint rather than a harmless default. During competition congestion, restoration may temporarily outrank adaptation, and a short-term, product-specific polyphenol strategy may be evaluated. Evidence for tart cherry, curcumin, and related products is heterogeneous [13,63,64,65]. The neutral curcumin trial and failure of green tea to augment the expected Nrf2 response underscore the danger of mechanism-by-association claims [13,14]. A varied diet rich in fruits and vegetables is not equivalent to chronic high-dose isolated antioxidant capsules.

Permissive signaling is not an instruction to tolerate unlimited fatigue. When residual fatigue threatens the next high-value stimulus, the dominant objective can legitimately shift from adaptation to restoration. This shift should be driven by a prespecified outcome hierarchy, not by retrospective reinterpretation of an intervention result.

### 4.3. Restoration

The restoration window becomes dominant when loss of function after damaging, congesting, or mechanically demanding work limits the next meaningful exposure. The objective is to restore force, range of motion, tissue tolerance, sleep, appetite, or readiness, not to erase every sign of training stress.

Adequate daily protein intake and distribution support remodeling and training adaptation [66,67,68]. They are best treated as foundational eligibility conditions, not evidence of a discrete restoration window. The cited literature does not establish that increasing protein specifically during a restoration state accelerates force or readiness independently of correcting inadequate intake. Creatine is similarly a saturation-dependent, block-scale strategy with established evidence for repeated high-intensity capacity, strength, and training adaptation [7,53]. It may be studied in high-load or rehabilitation blocks, but should not be presented as an acute recovery agent without a directly measured restorative endpoint. A morphology-focused synthesis distinguishes measured hypertrophy from body-composition and mechanistic surrogates [69].

Connective-tissue claims require even tighter boundaries. The frequently cited gelatin-plus-vitamin C experiment was a small crossover study in eight healthy men. Its human outcome was circulating procollagen type I *N*-terminal propeptide, a systemic bone/collagen-turnover marker that is not tendon-specific; the ligament findings came from engineered tissue exposed to participant serum [70]. It is a mechanistic proof of concept, not direct evidence that pre-loading ingestion improves tendon structure, symptoms, return to sport, or injury prevention. Collagen-derived peptides paired with mechanical loading remain plausible, but practical benefit and superiority of one schedule require direct trials [70,71].

Evidence for *n*-3 PUFA is likewise limited [72,73,74,75]. A randomized trial in healthy young women reported less muscle-volume loss during two weeks of unilateral immobilization after a four-week preload [73]. Without a matched normally loaded stratum, the trial supports efficacy within that disuse model but does not estimate an *n*-3 PUFA × disuse interaction. It should not be generalized to injury healing, routine post-exercise recovery, rehabilitation performance, or other populations without direct evidence.

Some polyphenol-rich products may improve short-term functional recovery after muscle-damaging exercise [63,64,65], but product identity, dose, exercise model, and outcome matter, and routine exposure must be judged against the adaptation objective. Restoration should not become a permanent philosophy of suppressing discomfort. The decision threshold is whether recovery of function is necessary to protect the next high-value exposure; total daily intake and protein distribution remain more defensible foundations than a narrowly defined post-exercise “anabolic window” [50,76].

### 4.4. Resilience

The resilience window concerns tolerance of the training and competition ecosystem rather than a single bout. It can become dominant during travel, sleep restriction, heat exposure, heavy camps, fixture congestion, gastrointestinal vulnerability, and periods of elevated illness risk. Strain- and product-specific probiotics and clinically indicated micronutrient correction are candidate strategies, but relevant outcomes are illness burden, gastrointestinal tolerance, and training availability rather than an assumed acute performance effect [77,78,79,80,81].

Probiotics are not generic acute travel-day interventions. Use should reproduce the genus, species, strain, dose, viability, product, and lead-in period used in supporting trials—often several weeks—rather than assume a class effect from starting any product at departure [77,81]. The athlete microbiome can differ from that of more sedentary populations, but descriptive differences do not establish an intervention target [82].

Bovine colostrum requires a more cautious classification. In the cited rugby trial, eight weeks of supplementation improved vertical-jump height but did not reduce reported illness incidence or change the measured immune and inflammatory markers [15]. That study supports neither immune resilience nor preferential use in a high-risk state. Colostrum remains, at most, a hypothesis-generating candidate for a product-specific resilience trial.

Resilience studies are vulnerable to uninformative null results when baseline risk is low. A probiotic is unlikely to demonstrate fewer illness days when incidence is minimal, and a micronutrient cannot correct a limitation that is absent [80]. Baseline vulnerability should therefore be specified before enrollment, not invoked after a null finding. Conversely, biochemical deficiency correction is a clinical management question and should follow compound-specific medical criteria rather than a training window alone.

Resilience widens the performance construct. Preventing missed training, reducing gastrointestinal disruption during competition, or preserving sleep and appetite during travel can improve a season despite no change in a laboratory time trial. Risk-targeted strategies should be withdrawn or deprioritized when the risk resolves, objective monitoring shows no benefit, or burden and cost exceed their contribution to availability.

## 5. Falsifiable Propositions and Prospective Validation

### 5.1. Four Propositions

The following propositions specify testable predictions for context, temporal allocation, pretreatment state, and complete-policy utility.

**P1**—For a fixed, fully specified, evidence-eligible intervention strategy, the treatment effect on the primary athlete-centered outcome will differ by at least a prespecified meaningful interaction margin and favor the target over the non-target window or context. The primary estimand is the intervention × window/context interaction contrast, coded so that positive values favor the target context. P1 is refuted for the tested strategy and outcome when the upper bound of the confidence interval lies below the prespecified meaningful interaction margin; this includes smaller-than-meaningful, negligible, and non-target-favoring effects. A confidence interval that spans the meaningful margin remains inconclusive.

**P2**—Temporal allocation. For an intervention whose kinetics permit meaningful schedule manipulation, exposure aligned with the target biological event will improve the primary outcome more than the same cumulative exposure delivered distally or off-window, by at least the prespecified smallest effect of interest and without unacceptable constraint costs. The target event, effective exposure period, schedules, adherence, washout, and carryover must be defined before allocation. P2 is refuted for the tested intervention, schedule, and outcome when a precise aligned-versus-distal contrast is smaller than the prespecified meaningful effect or favors distal timing. Failure to separate biological exposure is a failed manipulation, not a test of P2.

**P3**—Bottleneck/state dependence. Benefits will increase in the prespecified direction as an independently measured pretreatment limitation or risk state increases. The moderator must be measured before treatment and must not be defined from the eventual response. The primary estimand is the intervention × baseline-state interaction, preferably with the moderator retained continuously. P3 is refuted for the tested intervention, state variable, and outcome when an adequately precise interaction is negligible or directionally opposite, or when benefit is equal or larger when the proposed bottleneck is absent.

**P4**—Policy utility. A locked NPX-guided allocation policy will either improve the prioritized athlete-centered outcome by at least the smallest effect of interest or preserve it within a prespecified non-inferiority margin while reducing exposure, burden, cost, or adverse effects relative to standardized evidence-based use. Products, eligibility thresholds, assignment rules, outcome hierarchy, and exit rules must be locked before randomization. P4 is refuted when the policy is inferior or when an adequately precise comparison excludes both the prespecified superiority threshold and the non-inferiority-plus-efficiency criterion; failure caused by insufficient precision is inconclusive.

For P4, ‘standardized evidence-based use’ denotes an active, prospectively documented comparator policy using the same product-quality and safety gates, candidate products, evidence-supported dose ranges, clinical eligibility criteria, foundational diet/training support, monitoring intensity, and follow-up as the NPX arm. It may include ordinary individualized dosing, conventional event timing, loading/maintenance, and clinically indicated initiation or discontinuation; such practices are not exclusive to NPX. The experimental difference is the additional locked NPX mapping from measured objective, context, and state to functional-window assignment and threshold-triggered allocation or exit decisions. Both arm-specific decision rules and the situations in which their actions differ must be specified before randomization. For example, both arms could permit an individually tolerated caffeine protocol before designated priority sessions, while the NPX arm additionally applies a prespecified objective/state rule for withholding or reallocating exposure; the trial must document that the policies produce the intended decision contrast. If evidence-based practice already implements the same rules and the policies do not differ, P4 has no separable intervention to test. A deliberately suboptimal schedule, forced unnecessary exposure, or withdrawal of indicated treatment is not an acceptable comparator.

Table 3 specifies the directional prediction, primary estimand, and refutation rule for each proposition.

Trade-off is an outcome-classification rule rather than a universal biological proposition. A trade-off is present only when a prespecified benefit threshold and a prespecified constraint/harm threshold are crossed in opposite directions. A mechanistic biomarker change without an athlete-centered benefit or cost is insufficient to classify the result as a practical trade-off.

### 5.2. Validation Architecture and Design Requirements

NPX trials should distinguish component tests from policy tests. Component tests estimate P1–P3 through a prespecified intervention-by-context, aligned-versus-off-window timing/schedule, or intervention-by-state contrast. Policy tests evaluate P4 by comparing complete, locked allocation strategies. Evidence for one component interaction does not validate the whole NPX policy.

Support and refutation are local to the prespecified intervention or policy, population, comparator, context or schedule, state variable, and outcome. Support for P1–P3 establishes, at most, the tested component rule; support for P4 establishes, at most, the tested policy under its implementation conditions. A precise negative result requires rejecting or restricting that rule or policy within this scope, rather than claiming rejection of every possible NPX strategy. Conversely, a positive result does not validate untested products, windows, populations, or policies. The tested claim must remain fixed after the results are known; relabeling the target window or bottleneck cannot rescue a refuted rule. Replicated tests across independently specified settings would be needed to assess the broader usefulness and limits of the allocation approach.

**Figure 2:**
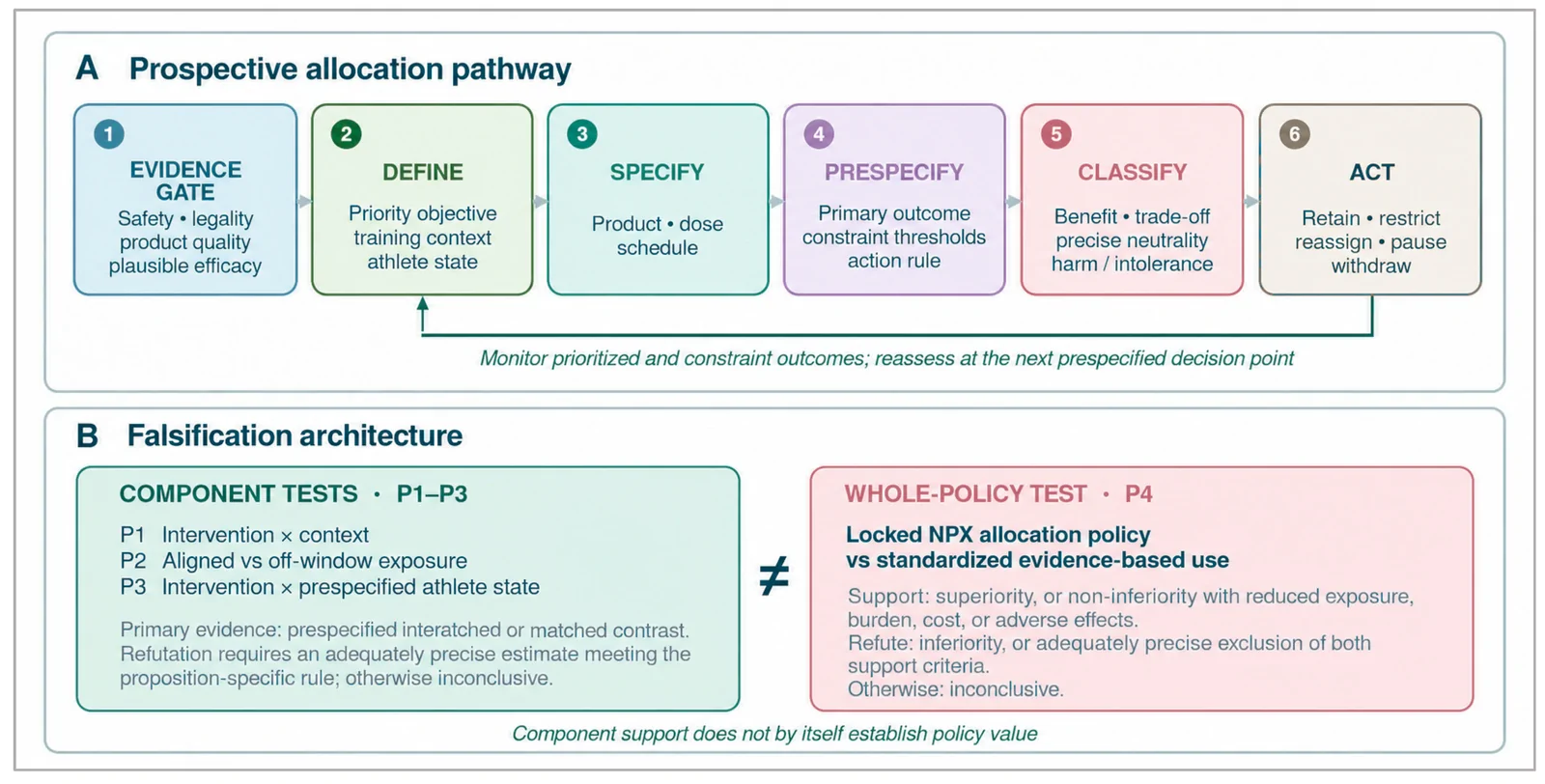
NPX allocation and falsification architecture. (**A**) A strategy enters NPX only after satisfying safety, legality, product quality, and plausible efficacy requirements. The priority objective, training context, athlete state, product, dose, schedule, athlete-centered primary outcome, constraint thresholds, and action rule are specified before exposure. The observed pattern is classified as benefit without unacceptable constraint, benefit with a trade-off, precise neutrality/context invariance, or harm/intolerance; insufficiently precise results remain inconclusive. This classification guides retention, restriction, reassignment, pausing, withdrawal, or further testing. (**B**) P1–P3 test individual allocation rules using prespecified interaction or matched contrasts, whereas P4 compares a locked NPX-guided policy with standardized evidence-based use. Refutation requires an adequately precise estimate meeting the proposition-specific rule; imprecise results are inconclusive.

The strongest component designs hold product, dose, cumulative exposure, adherence, and background training constant while manipulating the allocation variable. Examples include targeted versus inverted allocation of an acute ergogenic aid across high- and low-priority sessions; active versus placebo crossed with tendon loading versus matched rest; a standardized product crossed with high- versus normal-stress microcycles; and active versus placebo crossed with disuse versus normal loading. The complete policy requires a pragmatic randomized comparison between NPX-guided allocation and standardized evidence-based use. Supplementary Table S3 specifies prospective designs for eight exemplar tests. Figure 2 separates the prospective allocation pathway from the component and whole-policy tests used to evaluate it.

External load, internal load, intensity distribution, prior exercise, sleep, background diet, habitual supplement exposure, and adherence should be standardized or measured sufficiently to establish that the intended stress and exposure occurred [47,48,83]. Athlete state should be characterized with attention to training status, sex-related physiology where relevant, menstrual-cycle and hormonal-contraceptive status, energy availability, illness and injury status, gastrointestinal history, habitual diet, and micronutrient status [84,85]. These variables are candidate moderators, not licenses for universal subgroup protocols. Related systems monitoring emphasizes serial, phenotype-anchored biomarker panels as a proposed measurement approach [86].

Illustrative athlete-centered endpoints include time-trial performance, power, decision quality, or session completion for performance exploitation; longitudinal performance or functional adaptation for adaptation-permissive signaling; force or functional recovery for restoration; and illness days, gastrointestinal symptoms, missed sessions, sleep, or appetite for resilience. Mechanistic readouts can examine causal coherence but cannot substitute for these practical outcomes.

Design duration must match intervention biology. Acute agents can use crossover designs when familiarization, washout, and carryover are adequate. Loading agents require parallel or repeated-block designs long enough to change tissue availability. Blinding should address sensory and physiological cues such as taste, color, paresthesia, gastrointestinal effects, or stimulant-related arousal. Products with contamination risk require appropriate quality assurance.

Claims about responders require repeated measurement and a comparator because measurement error, day-to-day variation, and regression to the mean can mimic heterogeneity [21,87,88]. Sample-size planning should target the interaction or between-policy contrast, not significance within separate subgroups. Repeated observations should be modeled with within-athlete correlation, and cluster designs should model team or site effects. Replication across laboratories, sports, products, and training phases is required before a window-specific rule becomes actionable.

## 6. Discussion

### 6.1. Principal Contribution and Interpretation

The central contribution of NPX is to treat context as part of the allocation decision without treating context as a post hoc explanation for every result. Established classifications identify products that are sufficiently safe, legal, characterized, and efficacious to consider [1,44]. NPX addresses the subsequent decision: whether a fully specified eligible strategy should be used now, for this objective and state, under an explicit outcome hierarchy and exit rule.

The absence of a D-coded claim in the present evidence map is central to this interpretation. NPX-specific allocation benefits and whole-policy utility remain unvalidated hypotheses; established ingredient efficacy cannot supply the missing allocation contrast. This distinction limits the conclusions to a proposed decision architecture and research agenda rather than an evidence-supported supplement prescription policy.

A compound may alter a pathway without changing performance, improve short-term comfort without improving longitudinal adaptation, or show benefit under high recovery pressure. A robust main effect with a precisely negligible interaction supports broad efficacy and argues against an additional allocation layer. A null main effect with a reproducible prespecified interaction may support conditional efficacy. An underpowered interaction remains inconclusive.

The framework deliberately separates four concepts that are often conflated: the intervention strategy is manipulable; the training context defines the task; athlete state is a pre-exposure potential moderator; and the outcome hierarchy defines success and constraint. This separation prevents a conceptual “three-way interaction” from being asserted when a study has estimated only a main effect or a two-way contrast.

### 6.2. Potential Benefits and Practical Implications

If supported by prospective trials, NPX could help concentrate exposure where it adds practical value, reduce unnecessary supplementation, and make performance–recovery–adaptation trade-offs explicit. These are anticipated benefits rather than demonstrated effects of the NPX policy. Acute agents would be evaluated at the session scale and loading agents at the block scale; protein adequacy remains foundational. Proximal versus distal antioxidant exposure, collagen timing, preferential benefit of *n*-3 PUFA under disuse, and immune-resilience allocation remain narrower hypotheses than popular practice often implies. Probiotic claims must be product- and strain-specific, and micronutrient correction requires a credible deficiency or inadequacy state [77,80,89].

Precision should not be confused with labeling an athlete a responder after one noisy observation. A candidate moderator must be measured reliably, linked plausibly to the target mechanism, and validated against an athlete-centered outcome [21,22,23,24,87,88]. The most credible allocation variables are often concrete states—documented deficiency, persistent inadequate intake, disuse, repeated gastrointestinal problems, or a defined period of elevated illness risk—rather than a single fluctuating biomarker. Decision-linked biomarker frameworks likewise connect kinetics, sampling matrix, and the specific monitoring decision [90].

Digital measures may eventually improve state estimation. Wearable sensors can increase the temporal resolution of load, sleep, cardiovascular responses, temperature, and selected biochemical signals, while multi-omic studies demonstrate coordinated, time-dependent exercise responses [91,92,93]. Artificial intelligence and digital-twin concepts may assist integration, but current evidence does not justify autonomous nutraceutical prescription; measurement quality, mechanism, prospective validation, and human oversight remain essential [94,95].

### 6.3. Potential Risks and Implementation Costs

Allocation does not remove product-related risk. Caffeine can impair sleep or increase anxiety, sodium bicarbonate can provoke gastrointestinal symptoms, and supplement contamination can expose athletes to prohibited substances [1,6,56]. Chronic high-dose isolated vitamin C/E exposure has attenuated selected exercise-induced molecular adaptations in human trials, although functional consequences have varied [59,60,61]. These risks require product-specific screening, tested tolerability, and monitoring of constraint outcomes; a plausible window cannot make an unsuitable strategy acceptable.

The proposed allocation layer also creates decision risks. Misclassifying the dominant objective, treating a noisy biomarker as a bottleneck, or overlooking loading and residual exposure could lead to unnecessary use or mistimed restriction. In particular, a withdrawal rule should not interrupt correction of a documented deficiency or prescribed medical care, and a loading strategy cannot be managed as though its biological effects ceased immediately after the last dose. Safety and clinical indications therefore take precedence over window assignment [1,7,80].

Monitoring, reassignment, and multiple decision thresholds add time, financial cost, and adherence burden. They could encourage false precision or distract from energy and nutrient adequacy, sleep, and training management. Prospective policy trials should measure adverse events, sleep and gastrointestinal costs, adherence, total exposure, and implementation burden alongside the primary outcome. A simpler policy should remain preferable if the additional allocation process produces no meaningful benefit or efficiency gain.

### 6.4. Strengths and Limitations

The strengths of this review are its integration of molecular, nutritional, training-load, recovery, and precision perspectives; separation of evidence eligibility from allocation; explicit distinction between general efficacy and direct evidence of window dependence; and conversion of a conceptual proposal into refutable propositions, estimands, and policy tests. The framework is conservative: it prioritizes dietary adequacy, treats weakly supported strategies conditionally, and permits a simpler context-invariant model to outperform NPX.

Several limitations remain. This is a structured narrative review, not a systematic review. Retrieval was iterative; formal risk-of-bias and certainty grading were not performed; numerical screening counts and a prospectively registered audit trail were unavailable; and selection bias cannot be excluded. Qualitative selection, source weighting, claim definition, and E/C/M coding can reflect author judgment, especially when findings are mixed or product and population boundaries are uncertain. The examples are representative rather than exhaustive, and the efficacy descriptors are not formal certainty ratings or independently reliability-validated classifications. None of the allocation claims mapped in this review met the direct-evidence criterion, even when general intervention efficacy was established. These limitations support cautious interpretation of NPX as a hypothesis-generating architecture and preclude claiming validated allocation benefits or prescriptive superiority.

The four windows were generated by the authors rather than by formal consensus or Delphi methods and have not been externally validated. They are overlapping decision states, not biologically isolated compartments, and assignment requires judgment. Biomarkers are imperfect proxies for longitudinal performance, function, health, or availability. Chronic loading and residual exposure blur temporal boundaries. Interindividual variability in training status, habitual diet, sex-related physiology, energy availability, genetics, microbiome, injury status, and environment limits deterministic prescription. Finally, an allocation framework adds burden. NPX earns value only if prospective tests show better outcomes, more efficient exposure, or clearer decisions than simpler evidence-based use.

## 7. Conclusions

Nutraceutical Periodization for eXercise (NPX) proposes a testable allocation policy for evidence-eligible strategies across performance, adaptation, restoration, and resilience priorities. In the present evidence map, no candidate strategy met the criterion for direct NPX allocation evidence; the framework remains hypothesis-generating. Prospective component and active-comparator policy trials must establish whether particular rules improve athlete-centered outcomes or preserve them with lower exposure, burden, cost, or harm. Support or refutation applies to the intervention or policy, population, context, and outcome tested.

## Figures and Tables

**Table 1:** Construct-by-construct comparison of NPX with established evidence-based and periodized sports-nutrition practice.

Decision Construct	Existing Evidence-Based Practice	NPX Formalization and Prospective Requirement	Result Indicating No Added NPX Value
Evidence eligibility	Safety, legality, product quality, formulation, and general efficacy are assessed from compound-specific evidence.	Inherited entry gate; only eligible strategies proceed to allocation.	Failure at the gate; NPX is not applied.
Dominant objective	Training phase and nutrition goals are commonly aligned.	One functional window and its primary endpoint are assigned before exposure; other objectives become constraints.	The intervention effect is equally useful across contexts, or assignment does not improve prediction.
Bottleneck	Athlete needs and deficiencies may guide practice.	A limitation or risk state is defined independently before treatment and linked to the proposed mechanism.	Benefit is equivalent, larger when the bottleneck is absent, or the proposed state cannot be measured reliably.
Intervention and temporal fit	Dose and conventional timing follow product-specific guidance.	Product, formulation, dose, onset, persistence, duration, adherence, washout, and carryover are specified as one strategy.	Exposure-matched schedules are equivalent within a prespecified margin, or biological exposure is not separable.
Outcome hierarchy and trade-off	Performance, recovery, health, and adverse effects are evaluated, often separately.	One athlete-centered primary outcome, explicit constraint outcomes, and optional mechanistic outcomes are prespecified with decision thresholds.	The additional hierarchy does not change interpretation or action; mechanistic data add no decision value.
Exit and reallocation	Strategies are reviewed periodically.	Retain, restrict, shift, pause, or withdraw according to a locked rule and a change in objective or state.	A simpler fixed strategy achieves the same outcome and burden.
Whole-policy evaluation	Individual products are usually compared with placebo or usual practice.	A locked NPX policy is compared with an independently specified active evidence-based policy; both may include conventional timing and individualization. The additional NPX rules and actual decision differences are documented.	No superiority, and no non-inferior effectiveness with a prespecified reduction in exposure, burden, or harm.

Note: The evidence-eligibility gate is inherited from established practice and is not an NPX innovation. NPX, Nutraceutical Periodization for eXercise.

**Table 2:** Prospective assignment rubric for the four NPX functional windows.

Window	Operational Definition and Required Pre-Exposure Inputs	Primary Endpoint and Principal Constraints	Deployment Scale and Reassignment Trigger
Performance exploitation	Immediate task output is the locked primary objective; predefined function and availability constraints are satisfied. Define task, session priority, prior exposure, tolerance, and time of day.	Task performance, decision quality, or session completion; constrain sleep, anxiety, gastrointestinal symptoms, body-mass consequences, and adverse events.	Session for acute agents; block for loading agents. Reassign when immediate output no longer dominates or costs exceed the target-task benefit.
Adaptation-permissive signaling	A longitudinal oxidative, mitochondrial, or tissue-remodeling endpoint is primary; neither measured functional loss nor prospective availability risk crosses the predefined constraint threshold.	Longitudinal performance or functional adaptation; mechanistic markers are supportive. Constrain excessive fatigue, injury risk, and inability to complete planned training.	Session to block. Action may be non-initiation, restriction, rescheduling, pausing, or withdrawal. Reassign when a locked function or availability constraint is crossed; account for residual exposure and medical indications.
Restoration	A measured post-exercise, injury-related, or disuse-related functional deficit crosses a predefined threshold and threatens the next priority exposure. Define required function and recovery deadline.	Recovery or preservation of function and readiness; constrain suppression of a prioritized adaptation, adverse effects, and unnecessary exposure.	Hours to days or a rehabilitation/disuse block. Exit when function is restored, loading resumes, or the next objective changes.
Resilience	A predefined prospective travel, environmental, gastrointestinal, illness, or availability-risk criterion is crossed before the corresponding functional loss. Define indicator, threshold, time horizon, and exit condition.	Illness or gastrointestinal burden, missed sessions, or preserved availability; constrain treatment burden, adverse effects, and unsupported class-wide inference.	Defined risk period with intervention-specific lead-in. Exit when the risk resolves; deficiency correction follows clinical and biochemical criteria.

Note: Each assignment requires a dated priority objective, justified pre-exposure function and risk thresholds, and a locked priority/tie-break rule. When windows overlap, the qualifying objective that takes precedence under that rule is dominant; the other objectives become constraints. Unresolved overlaps should be recorded. Assignment must not be revised after the intervention response is observed. These proposed criteria have not yet undergone external reliability validation.

**Table 3:** Falsifiable propositions of the NPX framework.

Proposition	Directional Prediction	Primary Estimand	Refutation Rule
P1—Window/context effect modification	A fixed strategy has a meaningfully larger effect in the prospectively targeted than non-target context.	Intervention × window/context interaction contrast.	Upper confidence bound below the prespecified meaningful target-context interaction margin, including a smaller-than-meaningful, negligible, or non-target-favoring effect; an interval spanning the margin is inconclusive.
P2—Temporal allocation	Target-aligned exposure exceeds exposure-matched distal/off-window use by the prespecified meaningful amount without unacceptable constraints.	Aligned-versus-distal treatment contrast, with exposure separation verified.	A precise difference below the smallest effect of interest, or equal/better performance with distal timing.
P3—Bottleneck/state dependence	Benefit increases with an independently measured pretreatment limitation or risk state.	Intervention × baseline-state interaction.	A precise negligible/opposite interaction, or equal/larger benefit when the bottleneck is absent.
P4—Policy utility	A locked NPX policy is superior on the primary outcome, or non-inferior with lower exposure, burden, cost, or harm.	Intention-to-treat between-policy contrast against a prospectively specified active evidence-based comparator with documented policy differences; two-part non-inferiority-plus-efficiency gate when applicable.	Inferiority, or an adequately precise result excluding both the superiority threshold and non-inferiority-plus-efficiency criterion.

Note: An imprecise nonsignificant result is inconclusive, not evidence of invariance. The smallest effect of interest, equivalence region, non-inferiority margin, and acceptable constraint thresholds must be justified before recruitment. Support or refutation applies to the specified tested intervention/policy, population, comparator, context/state, and outcome; it is not global validation or rejection of NPX.

## Data Availability

No new data were created or analyzed in this study. Data sharing is not applicable to this article.

## Supplementary Materials

The following supporting information can be downloaded at: https://www.mdpi.com/article/10.3390/nu18203337/s1: Supplementary Method S1, Structured-Narrative Search and Synthesis Procedures; Table S1, Search Architecture and Executable Database Strings; Table S2, Evidence Map for Representative NPX Allocations; Table S3, Eight Prospective Designs for Testing NPX; Table S4, NPX Trial Preregistration and Reporting Checklist.

## Abbreviations

The following abbreviations are used in this manuscript:

Abbreviation	Definition
AMPK	AMP-activated protein kinase
mTORC1	Mechanistic target of rapamycin complex 1
*n*-3 PUFA	Omega-3 polyunsaturated fatty acids
NPX	Nutraceutical Periodization for eXercise
Nrf2	Nuclear factor erythroid 2-related factor 2
PGC-1α	Peroxisome proliferator-activated receptor gamma coactivator 1-alpha
