# Supplementary material for: Nutraceutical Periodization for eXercise (NPX): Matching Bioactives to Training Load, Recovery Demand, and Adaptation Windows
Source: Nutrients. 2026 Oct 12;18(20):3337. doi: 10.3390/nu18203337 (PMC13708102; doi:10.3390/nu18203337)
Supplement: Supplementary file 1 [file nutrients-18-03337-s001.zip › nutrients-4605472-supplementary.pdf]

nutrients

MDPI

Supplementary Material

Contents: Supplementary Methods S1; Tables S1–S4. Reference numbers correspond to the reference list in the main article.

Supplementary Methods S1. Structured-Narrative Search and Synthesis Procedures

S1.1. Review Design, Scope, and Unit of Inference

The review was designed as a structured narrative review and theory-building synthesis, not as a systematic review or meta-analysis [16–19]. Its purpose was to integrate heterogeneous evidence on exercise-adaptation biology, intervention efficacy and kinetics, training context, recovery demand, athlete-state modifiers, and periodized nutrition, and then translate that synthesis into prospectively falsifiable propositions. The review does not claim exhaustive retrieval, pooled treatment effects, formal certainty grading, or article-level risk-of-bias judgments.

The population of primary interest comprised competitive athletes and exercise-trained adults. Evidence from healthy non-athlete adults was retained when needed to characterize pharmacokinetics, tissue exposure, mechanism, or a controlled exercise response not adequately isolated in athletes. Clinical, injured, immobilized, deficient, or otherwise vulnerable populations were used only to inform the corresponding state-dependent mechanism or boundary condition; such evidence was not treated as direct proof of efficacy in healthy athletes.

Interventions within scope were protein and amino acids, creatine, caffeine, dietary nitrate, sodium bicarbonate, beta-alanine, polyphenols and isolated antioxidants, n-3 polyunsaturated fatty acids (n-3 PUFA), probiotics, bovine colostrum, medically indicated micronutrient correction, and collagen-derived peptides. Adequate energy and carbohydrate availability were treated as foundational conditions rather than as nutraceuticals allocated to a functional window. One human exercise study of acute nutritional ketosis [11] was retained only to illustrate endpoint discordance and was not mapped as an NPX candidate strategy.

The inferential unit was an explicitly specified intervention strategy: product, formulation, dose, exposure schedule, and duration. Outcomes were assigned to one of three roles before interpretation: (i) one prioritized athlete-centered outcome, such as performance, training completion, functional recovery, illness days, gastrointestinal symptoms, or missed sessions; (ii) constraint outcomes representing potential opportunity costs, such as sleep disruption, gastrointestinal intolerance, adverse events, body-mass change, or impaired adaptation; and (iii) mechanistic outcomes used to test biological coherence. A mechanistic response alone was not considered evidence of practical benefit.

S1.2. Information Sources and Dual-Track Search Architecture

PubMed/MEDLINE, Web of Science Core Collection, and Scopus were searched iteratively during manuscript development, with the final update completed in August 2026. Backward reference checking and forward citation tracking were conducted from consensus statements, position stands, systematic reviews, mechanistic reviews, and influential

athlete studies. The review retained the month of the final update rather than day-level search timestamps; database-specific result counts were not retained because the process was iterative and was not organized as a PRISMA-style systematic-review search log.

Three concept blocks supported the framework search: (A) exercise or athlete context; (B) nutraceutical, supplement, bioactive, or named intervention; and (C) allocation, timing, training context, athlete state, or outcome. The primary framework search combined A AND B AND C. To reduce the risk that timing-oriented terminology would preferentially retrieve studies already favorable to the proposed framework, a second efficacy-and-safety search combined block A with block B and an evidence-type block containing *systematic review*, *meta-analysis*, *consensus*, *position statement*, and *position stand*, without requiring block C. Compound-specific searches replaced the broad intervention block with the relevant named intervention and retained the context, kinetic, state, or outcome terms appropriate to the question.

Table S1 reports the searchable fields, transparent reporting boundaries, and final framework and efficacy-anchor strings. The retained record does not establish a database-inception start date, a uniform English-only or multilingual eligibility rule, or a consistently applied publication-status filter. Searches therefore cannot be reported as from inception, unrestricted by language, or uniformly filtered by document type. Interface-specific filter logs were not retained. Peer-reviewed sources were prioritized for efficacy claims, and unreviewed claims and commercial material were excluded as specified in Section S1.3; this selection rule does not establish the filters applied to each historical query.

S1.3. Eligibility and Evidence Prioritization

Evidence was eligible when it: (i) addressed at least one intervention class within scope, apart from the single endpoint-discordance exemplar identified in Section S1.1 [11]; (ii) involved an exercise, training, competition, recovery, injury/disuse, illness-risk, or athlete-relevant physiological context; (iii) reported an athlete-centered, constraint, kinetic, exposure, or mechanism-linked outcome relevant to the framework; and (iv) informed general efficacy and safety, exposure time course, context or state dependence, responder heterogeneity, or a plausible trade-off among performance, recovery, adaptation, and health.

Priority was given to peer-reviewed consensus statements and position stands, systematic reviews and meta-analyses, randomized human trials, longitudinal training studies, and controlled mechanistic human experiments. Seminal animal, tissue, or cell evidence was retained only where necessary to establish biological plausibility unavailable from human research and was not used to override neutral or adverse human findings. Commercial or promotional material, unreviewed claims, duplicate publications without additional information, inadequately characterized multi-ingredient products, studies without a meaningful exercise- or athlete-state link, and reports that did not permit the intervention, exposure, comparator, or relevant outcome to be identified were not used to support efficacy claims.

This was not an independently duplicated systematic-review screen. Records were assessed iteratively against the eligibility rules during framework development. Numerical screening counts, article-level exclusion reasons, duplicate-removal counts, reviewer-agreement statistics, and adjudication logs were not retained; accordingly, none is reported and a PRISMA flow diagram is not presented.

The available review record does not identify which author performed each literature search, eligibility decision, qualitative appraisal, or data-organization task, or establish whether each task was conducted by one or several authors. A task-level role log and documented disagreement-resolution procedure are unavailable and have not been

https://doi.org/10.3390/nu18203337

reconstructed retrospectively. The collective author-contribution statement denotes shared responsibility for the manuscript; it does not demonstrate independent duplicate procedures. This reporting limitation prevents independent reconstruction of the author-level workflow.

S1.4. Qualitative Appraisal and Evidence Coding

Formal risk-of-bias scoring and certainty grading were not undertaken. Each source was instead appraised qualitatively for study design, population relevance, intervention and product characterization, exposure and temporal fit, proximity and validity of the outcome for the claim, and consistency with higher-level human evidence. Sample size and precision, adherence, blinding credibility, diet and training-load control, carryover or washout, ecological validity, and adverse-event reporting were considered when available. Greater interpretive weight was assigned to convergent systematic reviews and well-characterized randomized human studies with outcomes aligned to the claim. Mechanistic evidence was used to explain plausibility, not to rescue an efficacy claim contradicted by athlete-centered human outcomes.

Table S2 uses two separate codes:

- Efficacy status applies to an explicitly bounded product or intervention, population, comparator, and athlete-relevant outcome, rather than to an ingredient universally. E denotes convergent human comparative evidence of practical efficacy: a consistent evidence synthesis and/or independently replicated, adequately characterized comparative studies with athlete-centered outcomes support the defined claim without unresolved major contradictory findings. C denotes human comparative evidence relevant to the practical claim, but with limited replication, inconsistent findings, imprecision, product or protocol heterogeneity, or extrapolation beyond the tested population or outcome. M denotes a proposed practical use supported chiefly by mechanism, kinetics, preclinical findings, or human surrogate outcomes, without convincing comparative evidence for the corresponding athlete-centered benefit. A narrow task or product scope does not itself lower a code; the code concerns support for that exact scoped claim. Mechanistic changes cannot elevate a claim to E, and neutral or adverse athlete-centered results remain explicitly visible rather than being treated as evidence of efficacy.
- Allocation directness is classified as D only when a prospective human comparison estimates the named timing-, context-, or state-dependent allocation contrast. For a context or state claim, this requires the relevant intervention-by-context/state contrast rather than benefit within a single context or state; for a timing claim, it requires a prespecified between-schedule comparison with adequately comparable exposure. I denotes an allocation inferred from general efficacy, kinetics, mechanism, a study conducted in only one candidate context or state, or comparisons across different studies.

The two codes answer different questions. A D code means that the relevant allocation contrast is estimable, not that it is meaningfully non-zero or supports NPX. An E code indicates efficacy within a defined scope but does not validate NPX-guided allocation. None of the specific allocation claims mapped in Table S2 met D; all mapped placements are I. This is a statement about these claims, not the absence of timing or individualization evidence throughout sports nutrition. Borderline or conflicting efficacy findings warrant a conditional or hypothesis-generating interpretation with explicit uncertainty. These qualitative labels are not formal risk-of-bias or certainty grades, and independently replicated coding or inter-rater reliability is not claimed. Null and discordant findings remain negative or uncertain evidence and are not reclassified after the fact as proof that the wrong window had been studied.

https://doi.org/10.3390/nu18203337

S1.5. Narrative Synthesis and Proposition Development

Synthesis proceeded in five stages. First, recurring physiological tasks within periodized training were grouped into performance exploitation, adaptation-permissive signaling, restoration, and resilience. Second, each intervention class was mapped according to general efficacy, target mechanism, onset and persistence, loading or washout requirements, candidate athlete-state modifiers, athlete-centered outcomes, and plausible constraint outcomes. Third, evidence for general efficacy was separated from direct evidence that timing, window, or state changes efficacy. Fourth, null, mixed, and discordant results were examined without assuming that a timing mismatch explained them. Fifth, the incremental NPX allocation layer was stress-tested by specifying observations that would make a compound-level claim, or the framework-level policy, unnecessary or incorrect.

The main article therefore distinguishes component tests from policy tests. Component tests estimate a prespecified intervention-by-context, intervention-by-timing, or intervention-by-state contrast. Policy tests compare complete allocation strategies. A full intervention × functional-window × athlete-state interaction is required only when all three factors are crossed and the study is powered for that three-way estimand. Separate subgroup p values do not constitute an interaction test [87,88]. Prospective protocols should define the target population, intervention strategy, functional allocation window, athlete-state moderator, prioritized outcome, smallest effect of interest or equivalence/non-inferiority margin, constraint outcomes, and rejection criterion before recruitment.

Support or refutation applies to the prespecified intervention or policy, population, comparator, context or schedule, state variable, and outcome. A positive component result does not validate an untested policy, and a positive policy result does not validate all NPX strategies. A precise negative result rejects or restricts the tested rule or policy within its stated scope. The target window, bottleneck, and inferential claim cannot be relabeled after observing the result. Broader usefulness requires independent replication across specified settings.

S1.6. Reporting Boundary

The procedures above make the structured narrative synthesis inspectable at the level of concepts, final search fields, eligibility rules, and inferential logic. They do not provide a complete reproducible historical search or author-level workflow: task assignments, inception and language boundaries, filter logs, counts, and adjudication records are unavailable. The review is not prospectively registered and should not be represented as a systematic review. No PRISMA flow counts, exhaustive database yield, independent duplicate-screening statistics, formal risk-of-bias tables, or quantitative certainty grades are claimed. Qualitative selection and coding may reflect author judgment; together with the absence of direct evidence for the mapped rules, this limits conclusions to hypothesis generation and prospective testing.

Table S1. Search Architecture and Executable Database Strings

***Table S1A**. Sources, Fields, Update, and Reporting Limits*

Database/source	Search field or method	Final update	Interface limits and reporting boundary
PubMed/MEDLINE	Title/Abstract free-text fields	August 2026	Inception date, language eligibility, and uniform publication-status filters cannot be verified from the retained record. Interface-filter logs, day-level timestamps, and result counts are unavailable. Final strings are reporting templates, not a complete historical log.
Web of Science Core Collection	Topic field (TS=)	August 2026	Same unverified inception, language, and publication-filter boundaries. Interface-filter logs, day-level timestamps, and result counts were not retained.

https://doi.org/10.3390/nu18203337

Database/source	Search field or method	Final update	Interface limits and reporting boundary
Scopus	Title, abstract, and keyword field (TITLE-ABS-KEY)	August 2026	Same unverified inception, language, and publication-filter boundaries. Interface-filter logs, day-level timestamps, and result counts were not retained.
Backward citation tracking	Reference lists of eligible anchor papers	Through August 2026	Not applicable—not a database query or systematic-review citation-chasing log.
Forward citation tracking	Cited-by records for eligible anchor papers	Through August 2026	Not applicable—not a database query or systematic-review citation-chasing log.

Table S1B*. Framework Searches: Exercise/Athlete × Intervention × Allocation/Context/State*

PubMed/MEDLINE

(exercis*[Title/Abstract] OR athlete*[Title/Abstract] OR sport*[Title/Abstract] OR training[Title/Abstract] OR competition[Title/Abstract] OR "physical performance"[Title/Abstract])
AND
(supplement*[Title/Abstract] OR "dietary supplement"[Title/Abstract] OR "dietary supplements"[Title/Abstract] OR nutraceutical*[Title/Abstract] OR bioactive*[Title/Abstract] OR protein[Title/Abstract] OR proteins[Title/Abstract] OR "amino acid"[Title/Abstract] OR "amino acids"[Title/Abstract] OR creatine[Title/Abstract] OR caffeine[Title/Abstract] OR nitrate[Title/Abstract] OR "sodium bicarbonate"[Title/Abstract] OR bicarbonate[Title/Abstract] OR "beta-alanine"[Title/Abstract] OR polyphenol*[Title/Abstract] OR antioxidant*[Title/Abstract] OR "omega-3"[Title/Abstract] OR "n-3 polyunsaturated fatty acid"[Title/Abstract] OR "n-3 polyunsaturated fatty acids"[Title/Abstract] OR probiotic*[Title/Abstract] OR "bovine colostrum"[Title/Abstract] OR micronutrient*[Title/Abstract] OR iron[Title/Abstract] OR "vitamin D"[Title/Abstract] OR collagen[Title/Abstract] OR gelatin[Title/Abstract])
AND
(timing[Title/Abstract] OR "nutrient timing"[Title/Abstract] OR periodization[Title/Abstract] OR periodisation[Title/Abstract] OR "biological window"[Title/Abstract] OR "biological windows"[Title/Abstract] OR exposure[Title/Abstract] OR onset[Title/Abstract] OR persistence[Title/Abstract] OR washout[Title/Abstract] OR carryover[Title/Abstract] OR session*[Title/Abstract] OR microcycle*[Title/Abstract] OR mesocycle*[Title/Abstract] OR "training block"[Title/Abstract] OR "training blocks"[Title/Abstract] OR adaptation[Title/Abstract] OR signaling[Title/Abstract] OR signalling[Title/Abstract] OR recovery[Title/Abstract] OR restoration[Title/Abstract] OR resilience[Title/Abstract] OR injury[Title/Abstract] OR illness[Title/Abstract] OR travel[Title/Abstract] OR heat[Title/Abstract] OR sleep[Title/Abstract] OR "precision nutrition"[Title/Abstract] OR responder*[Title/Abstract] OR heterogeneity[Title/Abstract] OR "training load"[Title/Abstract] OR "training loads"[Title/Abstract] OR "athlete state"[Title/Abstract] OR "athlete states"[Title/Abstract] OR "energy availability"[Title/Abstract] OR "recovery demand"[Title/Abstract] OR "recovery pressure"[Title/Abstract])

Web of Science Core Collection

TS=(exercis* OR athlete* OR sport* OR training OR competition OR "physical performance")
AND

TS=(supplement* OR "dietary supplement" OR "dietary supplements" OR nutraceutical* OR bioactive* OR protein OR proteins OR "amino acid" OR "amino acids" OR creatine OR caffeine OR nitrate OR "sodium bicarbonate" OR bicarbonate OR "beta-alanine" OR polyphenol* OR antioxidant* OR "omega-3" OR "n-3 polyunsaturated fatty acid" OR "n-3 polyunsaturated fatty acids" OR probiotic* OR "bovine colostrum" OR micronutrient* OR iron OR "vitamin D" OR collagen OR gelatin)
AND
TS=(timing OR "nutrient timing" OR periodization OR periodisation OR "biological window" OR "biological windows" OR exposure OR onset OR persistence OR washout OR carryover OR session* OR microcycle* OR mesocycle* OR "training block" OR "training blocks" OR adaptation OR signaling OR signalling OR recovery OR restoration OR resilience OR injury OR illness OR travel OR heat OR sleep OR "precision nutrition" OR responder* OR heterogeneity OR "training load" OR "training loads" OR "athlete state" OR "athlete states" OR "energy availability" OR "recovery demand" OR "recovery pressure")

Scopus

TITLE-ABS-KEY(exercis* OR athlete* OR sport* OR training OR competition OR "physical performance")
AND
TITLE-ABS-KEY(supplement* OR "dietary supplement" OR "dietary supplements" OR nutraceutical* OR bioactive* OR protein OR proteins OR "amino acid" OR "amino acids" OR creatine OR caffeine OR nitrate OR "sodium bicarbonate" OR bicarbonate OR "beta-alanine" OR polyphenol* OR antioxidant* OR "omega-3" OR "n-3 polyunsaturated fatty acid" OR "n-3 polyunsaturated fatty acids" OR probiotic* OR "bovine colostrum" OR micronutrient* OR iron OR "vitamin D" OR collagen OR gelatin)
AND
TITLE-ABS-KEY(timing OR "nutrient timing" OR periodization OR periodisation OR "biological window" OR "biological windows" OR exposure OR onset OR persistence OR washout OR carryover OR session* OR microcycle* OR mesocycle* OR "training block" OR "training blocks" OR adaptation OR signaling OR signalling OR recovery OR restoration OR resilience OR injury OR illness OR travel OR heat OR sleep OR "precision nutrition" OR responder* OR heterogeneity OR "training load" OR "training loads" OR "athlete state" OR "athlete states" OR "energy availability" OR "recovery demand" OR "recovery pressure")

***Table S1C**. Efficacy-and-Safety Anchor Searches: Exercise/Athlete × Intervention × Evidence Type*

These searches deliberately omit timing/context block C. Their purpose is to retrieve higher-level efficacy and safety evidence that could contradict, qualify, or render unnecessary an allocation hypothesis.

PubMed/MEDLINE

(exercis*[Title/Abstract] OR athlete*[Title/Abstract] OR sport*[Title/Abstract] OR training[Title/Abstract] OR competition[Title/Abstract] OR "physical performance"[Title/Abstract])
AND
(supplement*[Title/Abstract] OR "dietary supplement"[Title/Abstract] OR "dietary supplements"[Title/Abstract] OR nutraceutical*[Title/Abstract] OR bioactive*[Title/Abstract] OR protein[Title/Abstract] OR proteins[Title/Abstract] OR "amino acid"[Title/Abstract] OR "amino acids"[Title/Abstract] OR creatine[Title/Abstract] OR caffeine[Title/Abstract] OR nitrate[Title/Abstract] OR "sodium bicarbonate"[Title/Abstract] OR bicarbonate[Title/Abstract] OR "beta-alanine"[Title/Abstract] OR polyphenol*[Title/Abstract] OR antioxidant*[Title/Abstract] OR "omega-3"[Title/Abstract] OR "n-3 polyunsaturated fatty acid"[Title/Abstract] OR "n-3 polyunsaturated fatty acids"[Title/Abstract] OR probiotic*[Title/Abstract] OR "bovine colostrum"[Title/Abstract] OR micronutrient*[Title/Abstract] OR iron[Title/Abstract] OR "vitamin D"[Title/Abstract] OR collagen[Title/Abstract] OR gelatin[Title/Abstract])
AND
("systematic review"[Title/Abstract] OR meta-analysis[Title/Abstract] OR consensus[Title/Abstract] OR "position statement"[Title/Abstract] OR "position stand"[Title/Abstract])

Web of Science Core Collection

TS=(exercis* OR athlete* OR sport* OR training OR competition OR "physical performance")
AND
TS=(supplement* OR "dietary supplement" OR "dietary supplements" OR nutraceutical* OR bioactive* OR protein OR proteins OR "amino acid" OR "amino acids" OR creatine OR caffeine OR nitrate OR "sodium bicarbonate" OR bicarbonate OR "beta-alanine" OR polyphenol* OR antioxidant* OR "omega-3" OR "n-3 polyunsaturated fatty acid" OR "n-3 polyunsaturated fatty acids" OR probiotic* OR "bovine colostrum" OR micronutrient* OR iron OR "vitamin D" OR collagen OR gelatin)
AND
TS=("systematic review" OR meta-analysis OR consensus OR "position statement" OR "position stand")

Scopus

TITLE-ABS-KEY(exercis* OR athlete* OR sport* OR training OR competition OR "physical performance")
AND
TITLE-ABS-KEY(supplement* OR "dietary supplement" OR "dietary supplements" OR nutraceutical* OR bioactive* OR protein OR proteins OR "amino acid" OR "amino acids" OR creatine OR caffeine OR nitrate OR "sodium bicarbonate" OR bicarbonate OR "beta-alanine" OR polyphenol* OR antioxidant* OR "omega-3" OR "n-3 polyunsaturated fatty acid" OR "n-3 polyunsaturated fatty acids" OR probiotic* OR "bovine colostrum" OR micronutrient* OR iron OR "vitamin D" OR collagen OR gelatin)

https://doi.org/10.3390/nu18203337

AND

TITLE-ABS-KEY("systematic review" OR meta-analysis OR consensus OR "position statement" OR "position stand")

Table S1D. *Compound-Specific Substitution Rules*

Search purpose	Executable architecture	Use and interpretation
Compound-specific context search	Copy the relevant database framework string and replace the complete intervention block with one named-intervention expression listed below; retain the context/state terms relevant to the prespecified question.	Identifies timing-, state-, and training-context evidence for one specified intervention.
General efficacy/safety anchor	Copy the relevant database efficacy-anchor string and replace the complete intervention block with one named-intervention expression.	Prevents timing-oriented terminology from excluding neutral, adverse, or context-invariant efficacy evidence.
Kinetics/mechanism extension	Exercise/athlete block AND named-intervention expression AND (pharmacokinetic* OR bioavailability OR onset OR half-life OR washout OR carryover OR tissue concentration* OR mechanism*), using the database field syntax above.	Supports temporal plausibility and manipulation checks; it does not establish athlete-centered efficacy by itself.

Named-intervention expressions: protein OR proteins OR "amino acid" OR "amino acids"; creatine; caffeine; nitrate; "sodium bicarbonate" OR bicarbonate; "beta-alanine"; polyphenol* OR antioxidant*; "omega-3" OR "n-3 polyunsaturated fatty acid" OR "n-3 polyunsaturated fatty acids"; probiotic*; "bovine colostrum"; micronutrient* OR iron OR "vitamin D"; and collagen OR gelatin.

Table S1 note: Terms within a concept block are combined with OR; concept blocks are combined with AND. The strings reproduce the final common architecture. They do not constitute a prospectively registered, exhaustive search protocol, and no unretained counts or limits have been reconstructed.

Table S2. Evidence Map for Representative NPX Allocations

Table S2A. *Candidate Placement, Efficacy Status, Directness, and Citations*

Intervention strategy	Candidate window or decision role	Status/directness	Key citation(s)
Caffeine	Performance exploitation for a specified task or high-value session	E/I	[6,51]
Dietary nitrate	Performance exploitation for selected endurance or high-intensity tasks	C/I	[8,53,56]
Sodium bicarbonate	Performance exploitation when extracellular buffering is plausibly limiting	E/I	[55]
Creatine	Block-scale support for repeated high-intensity capacity, strength, and training adaptation	E/I	[7,52]
Beta-alanine	Loading block for tasks with substantial intracellular buffering demand	E/I	[9,54]
High-dose isolated vitamins C and/or E	Adaptation-permissive caution during blocks prioritizing oxidative or mitochondrial remodeling	C/I	[57–60]
Polyphenol-rich products	Conditional restoration during competition congestion or after tissue-damaging work	C/I	[13,14,62–64]
Protein and essential amino acids	Foundational adequacy and exercise-linked remodeling; not automatically a discrete restoration intervention	E/I	[65–67,75,76]
Gelatin or collagen-derived peptides with vitamin C	Mechanistic pre-loading candidate paired with tissue loading	M/I	[69,70]
n-3 PUFA	Conditional tissue-preservation candidate during immobilization or markedly reduced loading	C/I	[71–74]
Probiotics	Product- and strain-specific resilience course for a prespecified gastrointestinal or illness-risk outcome	C/I	[77,78,81]
Bovine colostrum	Hypothesis-generating resilience candidate; not an established immune-protection strategy	M/I	[15]

https://doi.org/10.3390/nu18203337

Intervention strategy	Candidate window or decision role	Status/directness	Key citation(s)
Targeted micronutrient correction	State-triggered correction of a clinically or biochemically established limitation	C/I	[80,89]

Codes: E, convergent comparative human evidence of practical efficacy for the defined product/intervention, population, comparator, and outcome; C, relevant human comparative evidence with limited replication, inconsistency, imprecision, or extrapolation; M, predominantly mechanistic, kinetic, preclinical, or surrogate support without convincing comparative athlete-centered benefit. A narrow task or product scope alone does not imply C. D, a prospective comparison estimates the specific allocation contrast; I, the allocation is inferred or lacks that comparison. None of the mapped claims met D; all placements are I, including those coded E for general efficacy. Efficacy codes do not validate the candidate window, and neutral or adverse findings are retained in Table S2B. These qualitative codes are not formal certainty grades or independently reliability-validated classifications.

Table S2B. *Temporal Features, Uncertainty, and Permissible Inference*

Intervention strategy	Temporal or state feature relevant to allocation	Uncertainty and permissible inference
Caffeine	Acute exposure can be aligned with a target task; pharmacokinetics, individual response, habitual use, and proximity to sleep may alter net value.	Acute ergogenic use is evidence-supported for several outcomes. Reserving doses for high-value sessions is a testable policy, not a demonstrated superior policy. Habitual-use attenuation should not be assumed without a direct comparison.
Dietary nitrate	Acute and short-course regimens have been studied; response varies with task, population, training status, and protocol.	Benefit is plausible for selected tasks, but a neutral trial in resistance-trained women limits class-wide claims [56]. High- versus low-priority session allocation has not been established.
Sodium bicarbonate	Pre-exercise exposure must balance buffering relevance against individualized gastrointestinal tolerance, sodium load, and medical contraindications.	Use can be evidence-supported for appropriate high-intensity tasks after tolerability testing. Broader NPX allocation superiority is untested.
Creatine	Tissue saturation develops across repeated dosing and persists beyond a single session; the relevant scale is usually a training block rather than an acute recovery period.	Established efficacy supports block-scale performance and adaptation use. Acute restoration, preferential benefit during energy deficit, and rehabilitation-specific superiority require direct outcomes and comparators.
Beta-alanine	Muscle carnosine accumulation requires loading; session-by-session timing is less informative than task demand, block objective, adherence, and washout.	Loading is evidence-supported for appropriate task durations. Superiority of NPX-guided block allocation over continuous evidence-based use remains untested.
High-dose isolated vitamins C and/or E	Existing trials used repeated daily exposure across training; they did not exposure-match proximal and distal schedules.	Chronic high-dose isolated regimens warrant caution when oxidative adaptation is prioritized. The claim that moving the same dose away from exercise prevents interference is untested; findings should not be generalized to antioxidant-rich foods. Related redox-periodization concepts are contextual background [61], not direct allocation evidence.
Polyphenol-rich products	Composition, dose, exposure duration, exercise-damage model, and time of outcome assessment vary materially among products and trials.	Some products may improve selected symptoms or functional recovery, whereas other trials are null. Short-term restoration use should remain product- and protocol-specific; universal recovery benefit or a universal anti-adaptive cost cannot be inferred.
Protein and essential amino acids	Total daily intake, quality, distribution, and exercise-linked meals jointly determine substrate availability; the magnitude of benefit depends strongly on baseline adequacy.	Adequacy is foundational. The cited literature does not establish an additional "restoration-window" effect on force or readiness independent of correcting inadequate intake. A morphology-focused synthesis distinguishes measured hypertrophy from mechanistic and body-composition surrogates [68].
Gelatin or collagen-derived peptides with vitamin C	A pre-loading schedule has mechanistic plausibility, but the commonly cited human endpoint was circulating PINP and the engineered-ligament result was ex vivo [69].	PINP is not tendon-specific. The evidence does not establish improved tendon structure, symptoms, return to sport, injury prevention, or superiority over another ingestion schedule.

https://doi.org/10.3390/nu18203337

Intervention strategy	Temporal or state feature relevant to allocation	Uncertainty and permissible inference
n-3 PUFA	Tissue incorporation requires pre-loading; one trial reported less muscle-volume loss during two weeks of unilateral immobilization after four weeks of supplementation [72].	The trial lacked a matched normally loaded stratum and therefore cannot estimate an n-3 PUFA × disuse interaction. Do not extrapolate to injury healing, routine recovery, or other populations without direct evidence.
Probiotics	Effects are genus-, species-, strain-, dose-, viability-, product-, outcome-, and course-specific; relevant studies commonly use a lead-in lasting weeks rather than travel-day initiation.	Match the proposed product and outcome to the supporting trial. A generic probiotic class effect and preferential benefit in a high-risk state require direct comparison.
Bovine colostrum	The cited rugby study used eight weeks of exposure and found improved vertical-jump height but no reduction in reported illness or change in measured salivary immune/inflammatory markers [15].	The study does not support immune resilience or preferential high-risk allocation. Colostrum may be studied prospectively but should not be represented as established immune protection.
Targeted micronutrient correction	Eligibility depends on a compound-specific clinical assessment, biochemical evidence where appropriate, dietary context, and planned reassessment.	Correcting a documented limitation is rational; benefit in replete athletes should not be assumed. A single fluctuating biomarker or low intake estimate does not by itself validate an NPX moderator threshold.

Table S2 note: The candidate windows are research classifications, not clinical or performance prescriptions. Every application remains subordinate to safety, legality, product quality, contamination control, medical contraindications, adequate energy and nutrient intake, and an evidence-based risk–benefit assessment [1–5].

Table S3. Eight Prospective Designs for Testing NPX

The designs below are exemplars, not completed trials. Each requires a protocol-specific ethical review, intervention safety assessment, product-quality verification, and prospective registration. The smallest effect of interest (SESOI), equivalence margin, or non-inferiority margin must be justified for the target population and outcome before recruitment; no sample size or effect magnitude is invented here. Support and refutation are specific to the tested intervention/policy, population, comparator, context/state, and outcome; none of these exemplar results alone can validate or reject the entire allocation approach.

S3.1. Does Allocating an Acute Ergogenic Aid to High-Priority Rather Than Low-Priority Sessions Improve the Block-Level Outcome?

Design element	Prospective specification
Main-article link	RQ2; component allocation test motivated by P1. Its primary contrast tests the allocation rule, not the P1 interaction or the P4 active evidence-based policy comparison.
Intervention and population	Test caffeine, dietary nitrate, or sodium bicarbonate in separate agent-specific studies in trained athletes whose block contains prospectively classified high- and low-priority sessions. Do not pool agents as one treatment.
Comparator	Experimental targeted allocation: active product before high-priority sessions and matched placebo before low-priority sessions. Experimental inverted allocation: the same number and total dose of active exposures before low-priority sessions and placebo before high-priority sessions. This matched contrast isolates allocation; the inverted schedule is not the P4 evidence-based comparator.
Minimum design	Randomized, blinded, counterbalanced repeated-block crossover when agent-specific washout and block comparability are credible; otherwise a randomized parallel-group design. The block objective and session-priority classification are locked before allocation.
Primary outcome and estimand	Select one block-level athlete-centered endpoint aligned with the block objective. Primary estimand: intention-to-treat between-policy difference in change from baseline. A session-level treatment × priority contrast is a secondary component estimand, not a substitute for the policy comparison.
Essential controls	Same product, formulation, dose, cumulative exposure, ingestion interval, sensory masking, adherence monitoring, and product-quality testing in both policies; standardized or measured pre-session nutrition, habitual intake, training load, time of day, sleep opportunity, and relevant medical contraindications.
Carryover and manipulation checks	Prespecify agent-specific washout and residual-exposure checks. Model period and sequence in crossover studies. Confirm ingestion, target exposure where feasible, and whether the planned difference in session allocation was achieved.
Sample-size basis	Power or precision calculation for the between-policy block-level contrast, using the prespecified SESOI, outcome variability, expected within-athlete correlation in a crossover, period effects, and attrition. Do not size the study only for an acute treatment main effect.

https://doi.org/10.3390/nu18203337

Design element	Prospective specification
Support, refutation, and inconclusive result	Support for this component allocation rule requires a targeted-versus-inverted benefit meeting the SESOI without unacceptable sleep, gastrointestinal, or other constraint costs. A confidence interval wholly within the prespecified equivalence region refutes incremental allocation value for this agent, schedule, and context. It does not test the complete P4 policy against active evidence-based practice. Imprecision or failed exposure separation is inconclusive.

S3.2. Does Antioxidant Exposure Proximal to Training Alter the Recovery–Adaptation Balance Relative to the Same Exposure Delivered Distally?

Design element	Prospective specification
Main-article link	RQ2; P2 (temporal alignment). Prespecify the target-aligned schedule and expected contrast direction; any recovery-adaptation trade-off requires the joint outcome criteria.
Intervention and population	One fully specified high-dose isolated vitamin C and/or E regimen during an adaptation-focused endurance or resistance-training block. Antioxidant-rich whole foods are outside this contrast.
Comparator	Three parallel arms: proximal active exposure, exposure-matched distal active exposure, and matched placebo. Product, cumulative dose, frequency, and total duration are identical in the two active arms.
Minimum design	Randomized, double-blind, parallel-group longitudinal training trial. The training stimulus, target adaptation, proximal and distal intervals, and biologically plausible exposure separation are defined before recruitment.
Primary outcome and estimand	One validated longitudinal adaptation outcome is primary. Primary estimand: proximal-versus-distal difference in change over the block; placebo anchors the direction and general effect of supplementation. If a trade-off claim is planned, one short-term recovery outcome is co-primary or hierarchically tested.
Essential controls	Training dose and completion, background diet and antioxidant intake, energy availability, sleep, additional recovery modalities, adherence, adverse events, and pathway-aligned mechanistic outcomes. Biomarkers remain explanatory and cannot replace the longitudinal adaptation endpoint.
Carryover and manipulation checks	Use a parallel design because training adaptations and repeated exposure can persist. Verify adherence and, where feasible, exposure separation; failure to separate biologically effective exposure is a failed manipulation rather than evidence against temporal specificity.
Sample-size basis	Base the calculation on the proximal-versus-distal adaptation contrast and its SESOI. If both recovery benefit and adaptation decrement are required for a trade-off classification, account prospectively for the joint directional decision and multiplicity.
Support, refutation, and inconclusive result	Temporal specificity is supported only if the schedule contrast meets the prespecified threshold. A recovery–adaptation trade-off requires both meaningful recovery improvement and meaningful adaptation decrement; a biomarker change alone is insufficient. Precise equivalence between schedules refutes proximity dependence for the tested regimen; inadequate precision is inconclusive.

S3.3. Does Aligning Collagen-Derived Peptide Exposure with Mechanical Loading Improve a Clinically Meaningful Tissue Outcome?

Design element	Prospective specification
Main-article link	RQ2; Proposition 2 (temporal alignment).
Intervention and population	A fully characterized collagen-derived peptide or gelatin product, with vitamin C specified if included, in athletes undergoing a standardized tendon-loading or rehabilitation program.
Comparator	Aligned active exposure before loading, the same active dose delivered off-window, and matched placebo, all with identical mechanical loading. An acute mechanistic substudy may separately cross active/placebo with loading/control loading if a supplement × load interaction is claimed.
Minimum design	Randomized, blinded, parallel-group clinical trial of sufficient duration for tissue function or symptoms to change. The loading prescription, alignment interval, off-window interval, cumulative exposure, and co-interventions are locked prospectively.
Primary outcome and estimand	A validated tendon- or tissue-specific functional, symptomatic, structural, or return-to-sport outcome. Primary estimand: aligned-versus-off-window difference in change. Circulating PINP and engineered-tissue responses may be secondary mechanistic outcomes but are not clinical endpoints.
Essential controls	Loading fidelity, rehabilitation exposure, dietary protein and vitamin C, analgesic or anti-inflammatory use, baseline tissue status, product batch and dose, adherence, adverse events, and assessor blinding.
Carryover and manipulation checks	Prefer a parallel design because tissue remodeling and rehabilitation effects persist. For an acute crossover mechanistic substudy, use a justified washout and demonstrate return to baseline before the next condition.
Sample-size basis	Size the clinical trial for the aligned-versus-off-window contrast on the chosen tissue-specific outcome. Size any mechanistic factorial substudy for the product × loading interaction, not for separate within-condition changes.
Support, refutation, and inconclusive result	Support requires a clinically meaningful aligned-versus-off-window benefit. A precise trivial contrast refutes timing superiority for that product and loading program. PINP change without improvement in the prespecified tissue outcome does not support clinical efficacy; an underpowered clinical contrast is inconclusive.

S3.4. Is the Effect of a Polyphenol-Rich Product Greater During a Congested or Tissue-Damaging Microcycle Than During a Normal-Load Microcycle?

Design element	Prospective specification
Main-article link	RQ2; Proposition 1 (context modification).
Intervention and population	One standardized polyphenol-rich product in trained athletes who complete both a prospectively defined high-stress microcycle and a normal-load microcycle. Product composition cannot be generalized to the whole class.
Comparator	Active product versus matched placebo within each microcycle context. The high-stress classification is based on planned external/internal load before recovery outcomes are observed.
Minimum design	Randomized, blinded 2 × 2 repeated-block design with counterbalanced treatment order where product washout and recovery-state reset are credible; otherwise a stratified parallel design containing both contexts.
Primary outcome and estimand	One athlete-centered recovery or training-availability outcome. Primary estimand: difference-in-differences, [(active − placebo) in the high-stress microcycle] − [(active − placebo) in the normal-load microcycle].
Essential controls	Product composition and dose, dietary polyphenols, training-load quantification, baseline soreness/function, sleep, energy and protein intake, other recovery modalities, adherence, and adverse events. Adaptation is a constraint outcome when repeated use spans a training block.
Carryover and manipulation checks	Prespecify product washout and criteria showing that recovery state returned to the planned baseline before crossover. Verify the intended difference in microcycle stress without defining context from post-treatment outcomes.
Sample-size basis	Base power or precision on the active × microcycle-context interaction, using repeated-measures correlation and the SESOI for the prioritized outcome. A calculation for an active-versus-placebo main effect is insufficient.
Support, refutation, and inconclusive result	A larger benefit in the prespecified high-stress context supports conditional allocation. Equivalent benefit in both contexts supports broad efficacy but not NPX-specific allocation. A precisely trivial interaction refutes context modification for the tested product; imprecision or failed context separation is inconclusive.

S3.5. Does a Pretreatment Recovery Bottleneck Modify the Benefit of Protein or Creatine?

Design element	Prospective specification
Main-article link	RQ3; Proposition 3 (bottleneck/state modification).
Intervention and population	Conduct separate agent-specific trials of protein or creatine in trained adults with and without a prospectively defined recovery-pressure state, such as controlled energy deficit, unusually high-volume loading, or rehabilitation. Background adequacy must be measured.
Comparator	Active strategy versus an agent-appropriate matched comparator in both bottleneck-present and bottleneck-absent strata. The bottleneck threshold is measured before treatment and cannot be defined from eventual response.
Minimum design	Randomized, controlled, stratified or factorial parallel design containing both state levels. If the recovery-pressure state cannot ethically be randomized, define it prospectively and address confounding in design and analysis.
Primary outcome and estimand	One agent-appropriate functional, lean-mass, rehabilitation, or training-completion outcome. Primary estimand: intervention × pretreatment recovery-pressure interaction.
Essential controls	Energy and protein intake, training or rehabilitation exposure, baseline body composition and function, formulation and dose, creatine loading status where relevant, adherence, gastrointestinal tolerance, body-mass change, and medical safety measures appropriate to the agent.
Carryover and manipulation checks	Use a parallel design when tissue saturation, body-composition change, or rehabilitation adaptation can persist. Verify the recovery-pressure manipulation or state independently of post-treatment outcomes.
Sample-size basis	Size the study for the interaction between intervention and pretreatment state, allowing for the expected state prevalence or planned stratum sizes, residual outcome variance, and attrition. Do not infer modification from significance in only one stratum.
Support, refutation, and inconclusive result	A meaningfully larger benefit in the bottleneck-present state supports the proposition. A precisely equivalent interaction, or equal/larger benefit in the bottleneck-absent state, refutes the proposed modifier for that intervention and outcome. Poorly separated strata or a wide interaction interval is inconclusive.

S3.6. Does a Prospectively Defined Gut–Immune Risk State Modify the Effect of a Product-Specific Probiotic or Bovine Colostrum Course?

Design element	Prospective specification
Main-article link	RQ3; Proposition 3 (bottleneck/state modification).
Intervention and population	Test a fully specified probiotic or bovine colostrum product in separate placebo-controlled trials. Include athletes in both prospectively defined high-risk and low-risk periods or strata; risk may involve travel, environmental heat, sleep disruption, prior gastrointestinal burden, or prior illness burden.
Comparator	Active product versus matched placebo within each risk stratum. For probiotics, genus, species, strain, dose, viability, product, storage, and lead-in are integral to the intervention.
Minimum design	Randomized, blinded parallel trial stratified by risk state, with both strata mandatory. Risk thresholds and primary endpoint are locked before allocation; a low-risk sample alone cannot test state modification.
Primary outcome and estimand	Choose one primary athlete-centered outcome: illness days, gastrointestinal symptom burden, or missed training sessions. Primary estimand: intervention × pretreatment risk-state interaction.

Design element	Prospective specification
Essential controls	Diagnostic or pathogen confirmation where feasible, exposure opportunity, travel/environmental conditions, sleep, diet, hygiene and illness-prevention co-interventions, training load, adherence, product viability, and adverse events. Immune biomarkers are mechanistic outcomes.
Carryover and manipulation checks	Respect product-specific lead-in, persistence, and washout. Prefer parallel groups when microbiome or immune effects may persist; use crossover only if full washout and stable risk periods can be justified. Confirm that the intended risk-state contrast existed independently of treatment.
Sample-size basis	Base the calculation on the interaction and the distribution of the selected outcome. For count, recurrent-event, or time-to-event outcomes, use an analysis-compatible variance/event-rate assumption from external evidence or pilot data and account for clustering by team or trip where applicable.
Support, refutation, and inconclusive result	A meaningfully larger benefit in the high-risk stratum supports state-dependent allocation. A precise trivial interaction refutes state dependence; an invariant overall benefit supports broad product-specific efficacy instead. Low event incidence, uncertain product viability, or inadequate risk separation makes the test inconclusive.

S3.7. Is n-3 PUFA More Protective During Disuse Than During Normal Loading?

Design element	Prospective specification
Main-article link	RQ3; Proposition 3 (bottleneck/state modification).
Intervention and population	A fully characterized n-3 PUFA regimen in an ethical controlled-disuse model or in a prospectively defined injury/reduced-loading cohort, with a matched normal-loading condition or stratum.
Comparator	Active n-3 PUFA versus matched placebo under both disuse/reduced loading and normal loading. The normal-loading comparator is mandatory to estimate preferential benefit under disuse.
Minimum design	Randomized 2 × 2 parallel design when loading state can be assigned ethically, or a prespecified stratified design with explicit confounding control when injury/disuse cannot be randomized.
Primary outcome and estimand	Preserved function or lean mass, selected prospectively. Primary estimand: [(n-3 PUFA − placebo) during disuse] − [(n-3 PUFA − placebo) during normal loading]. Molecular signaling or muscle-protein-synthesis outcomes are secondary.
Essential controls	Baseline n-3 status, dose and formulation, dietary n-3 intake, protein and energy intake, incorporation period, immobilization or loading fidelity, rehabilitation, adherence, body composition/function measurement quality, and bleeding or gastrointestinal adverse events.
Carryover and manipulation checks	Use a parallel design because tissue incorporation and disuse effects persist. Verify tissue exposure where feasible and quantify the loading-state contrast. A unilateral within-person model must account for systemic exposure and correlated limbs.
Sample-size basis	Size the trial for the n-3 PUFA × loading-state interaction on the athlete-centered primary outcome, incorporating correlation if limbs or repeated observations are used and allowing for attrition.
Support, refutation, and inconclusive result	Preferential preservation during disuse supports the proposition. A precisely trivial interaction refutes disuse specificity, even if a general main effect exists. Signaling changes without function or lean-mass preservation do not establish practical benefit; inadequate incorporation or state separation is inconclusive.

S3.8. Does a Locked NPX Allocation Policy Improve Athlete Outcomes or Preserve Them with Lower Exposure Burden?

Design element	Prospective specification
Main-article link	RQ4; Proposition 4 (incremental policy value).
Intervention and population	A complete NPX policy with locked eligibility gates, permitted products, product-specific dose/schedule rules, bottleneck thresholds, window-assignment rubric, outcome hierarchy, constraint thresholds, reassessment interval, and exit rules.
Comparator	Active, prospectively specified evidence-based policy using the same product-quality and safety gates, candidate products, evidence-supported dose ranges, clinical eligibility criteria, foundational diet/training support, monitoring intensity, and follow-up. Conventional individualization, event timing, loading/maintenance, and clinically indicated initiation/discontinuation are permitted. The added NPX objective/state-to-window mapping and threshold-triggered allocation/exit rules are the experimental difference; document both policies and the circumstances in which their decisions differ. Identical policies cannot test P4. Forced unnecessary exposure, deliberately suboptimal timing, or withdrawal of indicated treatment is not an acceptable comparator.
Minimum design	Pragmatic individual- or cluster-randomized parallel trial across a full training block or season. Cluster randomization is preferable where athletes share staff and contamination between policies is likely. The decision algorithm must be locked before randomization.
Primary outcome and estimand	One prioritized block- or season-level athlete-centered outcome. Primary estimand: intention-to-treat between-policy contrast. If parsimony is claimed, use a two-part gate: outcome non-inferiority followed by superiority for supplement exposures, adverse effects, or cost.
Essential controls	Same eligible products and allowed dose ranges, batch testing, clinical access, foundational nutrition support, co-interventions, monitoring contact, and adverse-event reporting opportunities. Lock both policy algorithms, their expected decision differences, override rules, and exit conditions before randomization. Record every allocation/non-use decision, override, adherence event, and deviation. Equal cumulative exposure is not required when reduced exposure is a prespecified policy objective.

https://doi.org/10.3390/nu18203337

Design element	Prospective specification
Carryover and manipulation checks	Use a parallel policy comparison because training, behavior, and product-loading effects may persist. Quantify policy fidelity, between-arm contamination, prohibited co-interventions, and whether the algorithms produce distinct decisions and exposure patterns. If the policies do not differ, no separable NPX policy effect has been tested.
Sample-size basis	Size for the between-policy primary estimand. Cluster designs require an intracluster-correlation and design-effect allowance; non-inferiority claims require a justified margin and adequate power for the non-inferiority gate, followed by the prespecified parsimony test.
Support, refutation, and inconclusive result	Support requires superiority by the prespecified meaningful threshold, or outcome non-inferiority plus a prespecified meaningful reduction in exposure, burden, adverse effects, or cost. Refutation requires inferiority or an adequately precise comparison excluding both prespecified success criteria; unacceptable registered constraint costs can overturn benefit. Imprecision or an unachieved policy contrast leaves utility uncertain and must be reported alongside intention-to-treat estimates. Support/refutation concerns this policy, population, setting, comparator, and outcome, not NPX globally.

General statistical note for Table S3: Interaction claims must be based on the interaction contrast itself, not on significance in one subgroup and non-significance in another [87,88]. Continuous state variables should remain continuous where possible; any threshold used operationally must be justified independently of treatment response. Repeated observations require models that account for within-athlete correlation and, where relevant, team or site clustering. Randomized comparisons should use an intention-to-treat estimand, with per-protocol analyses treated as sensitivity analyses. A nonsignificant but imprecise result is inconclusive. Post-treatment "responder" categories cannot define the moderator being tested.

Table S4. NPX Trial Preregistration and Reporting Checklist

The checklist is intended for component trials and whole-policy trials. "Preregister" means that the item is time-stamped before recruitment or, for a secondary analysis, before access to outcome data. "Report" means that the manuscript provides the prespecified item, the observed implementation, and any deviation. Items that genuinely do not apply should be marked "not applicable" with a reason rather than omitted.

Domain	Preregister before recruitment or outcome access	Report in the completed study
1. Study identity and version	□ Registry, protocol title, version, date, sponsor/funder, and analysis-plan location.	□ Registry identifier, accessible protocol and statistical-analysis-plan versions, amendments, and dates.
2. NPX question and proposition	□ Specify RQ/proposition, whether the study is a component or policy test, and the single causal question.	□ State the tested proposition without broadening it after results are known.
3. Target population and setting	□ Eligibility, sport/training status, sex/gender plan, age range, setting, competitive calendar, and relevant clinical exclusions.	□ Recruitment source, flow, baseline characteristics by arm/state, and external-validity boundaries [85,86].
4. Safety, legality, and product governance	□ Medical contraindications, anti-doping/legal review, contamination-control plan, batch verification, storage, and stopping responsibility.	□ Product source, batch/lot, independent testing where used, storage, deviations, adverse events, and any regulatory or anti-doping issue [1–5].
5. Intervention strategy	□ Product, formulation, dose, schedule, duration, loading/maintenance, and permitted adjustments. Specify non-initiation, restriction, rescheduling, pausing, or withdrawal as explicit actions when relevant; medical indications and dietary adequacy take precedence.	□ Exact intervention and intentional non-use actions delivered, fidelity, adherence, dose changes, interruptions, residual exposure, and contamination.
6. Comparator and treatment contrast	□ Placebo/no-treatment/active comparator or alternative policy; specify constants and differences. For P4, lock a credible active evidence-based policy that permits conventional timing and individualization; identify the additional NPX rules and expected decision differences.	□ Comparator delivered, masking, balanced co-interventions, policy decisions, and achieved treatment contrast. Report if both policies produced the same decisions.
7. Functional allocation window	□ Dated priority objective, scale, measured pre-exposure function/risk inputs, justified thresholds, start/end rules, and a locked priority/tie-break rule for overlapping windows. Record unresolved overlap; do not assign from treatment response.	□ Window assignments, durations, overlaps/unresolved cases, deviations, assessor agreement where measured, and sensitivity to thresholds. Reliability evidence does not establish outcome or policy utility.

https://doi.org/10.3390/nu18203337

Domain	Preregister before recruitment or outcome access	Report in the completed study
8. Athlete-state moderator or bottleneck	□ Measurement, timing, continuous scale, any threshold and its external justification; measure before treatment.	□ Distribution, measurement reliability, threshold performance, missingness, and confirmation that the state was not defined by response.
9. Temporal fit and exposure model	□ Expected onset, peak/effective exposure, persistence, loading, washout, and carryover; define aligned and off-window schedules.	□ Actual ingestion timing, exposure verification where feasible, washout adherence, residual exposure, and failed manipulations.
10. Primary athlete-centered outcome	□ One prioritized outcome, measurement method, time point, direction of benefit, and scoring rule.	□ Results for every prespecified time point with effect estimate, confidence interval, and absolute units.
11. Constraint outcomes	□ Prespecified sleep, gastrointestinal, symptom, adverse-event, body-mass, adaptation, burden, or cost constraints; justify the thresholds that can overturn benefit and medical overrides that prohibit withholding indicated care.	□ Every registered constraint result, including null/adverse findings, threshold crossings, medical overrides, and how these changed allocation or intentional non-use decisions.
12. Mechanistic outcomes	□ Mechanism, assay, sampling schedule, and role in the causal model; label as explanatory rather than athlete-centered unless justified.	□ Assay procedures and results without substituting a biomarker for an unmeasured practical outcome.
13. Estimand	□ Population, treatment condition, outcome, time point, handling of intercurrent events, and contrast (main effect, interaction, difference-in-differences, or policy effect).	□ Estimate the registered contrast directly; do not replace an interaction with separate within-group or subgroup tests [87,88].
14. Decision threshold	□ SESOI, equivalence region, non-inferiority margin, or superiority criterion, with empirical or stakeholder justification.	□ Confidence interval interpreted against the registered threshold; distinguish refutation/equivalence from imprecise inconclusiveness.
15. Sample-size justification	□ Expected variance/event rate, correlation, attrition, clustering, multiplicity, target power or precision, and calculation for the primary estimand.	□ Planned and achieved sample, exclusions, events, intracluster correlation where relevant, and consequences of lower precision.
16. Randomization and allocation concealment	□ Sequence generation, unit of randomization, stratification/blocking, concealment, and who can access the sequence.	□ Implementation, allocation failures, baseline imbalances, cluster recruitment timing, and deviations.
17. Blinding and placebo credibility	□ Who is blinded, placebo matching, emergency unblinding, and blinding assessment where informative.	□ Blinding implementation, unblinding, sensory differences, and credibility assessment without using it to exclude participants post hoc.
18. Carryover, washout, and period effects	□ Agent-specific washout, return-to-baseline rule, sequence/period model, and criterion for abandoning crossover.	□ Washout achieved, residual exposure, period/sequence estimates, and sensitivity analysis for carryover.
19. Training, diet, recovery, and environmental co-exposures	□ Variables to standardize or measure, including training load, energy/protein intake, habitual supplement use, sleep, travel, heat, and recovery modalities.	□ Achieved exposures by arm/context and how imbalance was addressed without data-driven covariate selection.
20. Adherence and manipulation checks	□ Adherence definition, measurement, exposure marker if available, and minimum criterion for a valid manipulation.	□ Adherence distributions, exposure separation, protocol fidelity, and explicit classification of a failed manipulation.
21. Statistical model	□ Model family, link, repeated-measures structure, random effects, clustering, covariates, period/sequence terms, and diagnostic checks.	□ Full model specification, effect estimates and intervals, diagnostics, convergence issues, and code/software version.
22. Missing data, intercurrent events, and analysis sets	□ Intention-to-treat strategy, missing-data assumptions and method, withdrawal, rescue treatment, illness/injury, and per-protocol sensitivity analysis.	□ Amount/reason/timing of missingness, intercurrent events by arm, primary analysis set, and registered sensitivity analyses.
23. Multiplicity and directional decisions	□ Outcome hierarchy, co-primary logic, family-wise or false-discovery control where needed, and joint criteria for trade-off claims.	□ All registered analyses and adjusted/unadjusted results as specified; do not select the favorable component of a joint claim.
24. Heterogeneity and subgroup analyses	□ Moderator analyses limited to prespecified baseline variables; retain continuous variables where possible.	□ Interaction estimates with intervals; label any unregistered subgroup or responder analysis exploratory [41,87,88].
25. Harms and stopping rules	□ Adverse-event definitions, severity/relatedness, surveillance interval, clinical escalation, and stopping boundaries.	□ Events and withdrawals by arm, denominators, relatedness assessment, stopping decisions, and absence of events without euphemism.
26. Policy fidelity and parsimony (policy trials)	□ Lock eligibility gates, both policy algorithms, objective/state thresholds, window priority/tie-break rules, non-use and override actions, exposure-burden/cost methods, and the non-inferiority-plus-efficiency sequence.	□ Every decision/non-use action and override, unresolved overlaps, both policies' fidelity, contamination, decision/exposure differences, harms/costs, and both inferential gates regardless of result.
27. Protocol deviations and post hoc analyses	□ Process for documenting and classifying deviations and the boundary between confirmatory and exploratory work.	□ Deviations with timing and reason; label all unregistered analyses and thresholds as exploratory.

Domain	Preregister before recruitment or outcome access	Report in the completed study
28. Transparency and reproducibility	□ Data dictionary, materials, statistical code, de-identification, sharing restrictions, conflicts of interest, and funder role.	□ Participant-level or suitably aggregated data where permissible, code, intervention materials, access conditions, conflicts, and funder influence.
29. Interpretation and rejection rule	□ Prespecify support/refutation/inconclusive patterns and their scope: intervention/policy, population, comparator, context/state, and outcome. Lock the claim; do not permit post-result relabeling.	□ Apply the registered rule locally; preserve null/discordant results and uncertainty. Do not rescue a negative rule with an unmeasured wrong window or infer global NPX validation/rejection.

Table S4 note: The checklist supplements, rather than replaces, discipline-appropriate trial-reporting standards. Sex- and gender-related design and reporting should follow current sport and exercise methodology guidance [85,86]. The statistical principles for individual-response and moderator claims follow references [41,87,88].

Abbreviations

D, direct allocation evidence; **E**, established efficacy within a defined scope; **GI**, gastrointestinal; **I**, indirect allocation evidence; **M**, mechanistic or hypothesis-generating evidence; **n-3 PUFA**, omega-3 polyunsaturated fatty acids; **NPX**, Nutraceutical Periodization for eXercise; **PINP**, N-terminal propeptide of type I collagen; **RQ**, research question; **SESOI**, smallest effect size of interest.

https://doi.org/10.3390/nu18203337
